# Chitosan-Gellan Gum Nanoparticles for Antituberculosis Drug Encapsulation: Synthesis, Characterization and In Vitro Investigation

**DOI:** 10.3390/polym18172139

**Published:** 2026-09-02

**Authors:** Yerkeblan Tazhbayev, Siu-Yin Cheung, Aldana Galiyeva, Miroslav Slouf, Libor Kostka, Tolkyn Zhumagaliyeva, Lyazzat Zhaparova, Arailym Daribay, Alibi Kaziyev

**Affiliations:** 1 Institute of Chemical Issues, Buketov Karaganda National Research University, Karaganda 100028, Kazakhstan; cheung.s8103@gmail.com (S.-Y.C.); zhumagalievat79@gmail.com (T.Z.); lyazzh@mail.ru (L.Z.); arailymdaribay@gmail.com (A.D.); 2 Institute of Macromolecular Chemistry, Czech Academy of Sciences, 162 00 Prague, Czech Republic; slouf@imc.cas.cz (M.S.); kostka@imc.cas.cz (L.K.); 3 National Scientific Centre of Phthisiopulmonology, Almaty 050010, Kazakhstan; alibilife228@gmail.com

**Keywords:** polyelectrolyte complexes, polysaccharides, anti-tuberculosis drug, isoniazid

## Abstract

Pulmonary delivery of anti-tuberculosis drugs has emerged as a promising alternative for increasing local drug concentrations whilst minimising side effects. Natural polymers—polysaccharides capable of forming polyelectrolyte complexes (PECs) through electrostatic interactions—show great promise in this field. In this study, mucoadhesive chitosan-gellan gum nanoparticles were developed for the controlled release of isoniazid using the polyelectrolyte complex coacervation method. Parameters such as the chitosan-to-gellan gum ratio and the medium pH were investigated to achieve suitable particle size, polydispersity, surface charge, drug loading and encapsulation efficiency. The morphology of the produced nanoparticles was determined using transmission electron microscopy (TEM) and nanoparticle tracking analysis (NTA), which confirmed the formation of nanoscale spherical particles. The mucoadhesive properties were tested on ovine lung tissue using fluorescence retention analysis, whilst in vitro drug release was studied under physiological conditions. Antimycobacterial activity against *Mycobacterium tuberculosis* H37Rv was assessed using the Mycobacteria Growth Indicator Tube (MGIT) system, followed by subculturing on Löwenstein–Jensen medium. The produced nanoparticles exhibited high mucoadhesion, maintaining prolonged retention on lung tissue. Drug release showed an initial burst followed by sustained release over 24 h. These results demonstrate that CSGG–INH nanoparticles possess sustained drug release, good mucoadhesion to the lungs and high antimycobacterial activity, supporting their further development as potential carriers for pulmonary delivery of anti-tuberculosis drugs.

## 1. Introduction

Tuberculosis (TB) is one of the leading causes of death from infectious diseases worldwide. According to the World Health Organization, approximately 10.7 million people were infected with tuberculosis in 2024, and 1.23 million people died from the disease [1]. The primary treatment for tuberculosis requires patients to take first-line anti-TB drugs such as isoniazid, rifampicin, pyrazinamide, and ethambutol [2]. The treatment drug isoniazid stands out as the leading and most effective choice because it demonstrates a remarkable ability to kill *Mycobacterium tuberculosis* (MTB) cells during the initial stage of treatment. The therapeutic effectiveness of isoniazid is limited by its rapid metabolic breakdown and its potential to cause systemic side effects such as hepatotoxicity [3,4]. Therefore, there is a great urgency to explore and develop effective methods for the delivery of anti-TB drugs such as isoniazid (INH).

Polymeric nanoparticles (NPs) are promising drug carriers due to their numerous advantages. It enhances the drug accumulation at the absorption site because of the small size of NPs, and a targeted drug delivery system can be achieved in order to minimize the toxicity to healthy tissues [5]. Using polysaccharides in the formation of polymeric NPs has extra benefits such as biodegradability, biocompatibility, non-toxicity and cost effectiveness. It not only improves the solubility of hydrophobic drugs but also allows for the controlled and sustained release of drugs over time [5,6].

The use of polysaccharide-based polyelectrolyte complexes (PECs) in the development of drug delivery systems has great potential. PECs are formed due to the electrostatic interaction between oppositely charged polymers [5,7,8,9,10]. This formation process is driven by an increase in entropy due to the release of counterions [11,12]. As the PEC formation involves ionic interactions, extra toxic or bio-incompatible cross-linking agents are unnecessary [7,13,14]. Furthermore, the properties of PEC drug delivery systems can be adjusted by various factors, including the type and ratio of polymers, charge density of polymers, polymer concentrations, pH, ionic strength of the medium and mixing methods [12,15,16].

PECs can be produced by polysaccharides such as chitosan and gellan gum. Chitosan (CS) is a biocompatible and cationic polysaccharide obtained through the deacetylation of chitin. The presence of chitosan in drug delivery systems enhances the drug permeation and the mucoadhesive property. Moreover, drug release is favored in an acidic environment due to the pH sensitivity of chitosan [17,18,19]. Gellan gum (GG) is a natural and anionic polysaccharide with gelation property [20]. The presence of gellan gum prevents the rapid release of drugs in the low-pH environment in the chitosan-gellan gum drug delivery systems [15,16].

Previous studies have demonstrated the potential of CSGG PEC systems for the encapsulation of antifungal agents, antibiotics, proteins and targeted therapeutic compounds [7,15,16,17]. However, most reported chitosan–gellan gum systems have been developed as hydrogels or gel-based delivery platforms, whereas studies on CSGG polyelectrolyte complex nanoparticles remain limited. Furthermore, CSGG PEC systems are not sufficiently studied as mucoadhesive carriers for delivery of antituberculosis drugs. Mucoadhesive nanoparticles have attracted considerable interest as potential carriers for inhalation delivery, since their prolonged retention on the respiratory tract mucosa can increase the local concentration of the drug, prolong its release, and reduce systemic side effects [21,22,23]. Chitosan is suitable for this purpose due to its mucoadhesive properties, while the use of gellan gum ensures the stabilization of the nanoparticles. Integrating these polysaccharides into a single PEC represents a promising approach to the development of nanocarriers with potential application in pulmonary drug delivery.

Despite the advances in PEC formulation, some important parameters, such as loading efficiency and nanoparticle yield, remain insufficiently investigated in previous works. Understanding these factors is key to optimizing the formulation of PEC-based nanoparticles to create efficient drug delivery systems. Therefore, in this work, we aimed at synthesizing and characterizing chitosan-gellan gum-based PEC nanoparticles for the encapsulation of isoniazid. We studied the effects of different formulation parameters, such as the chitosan-to-gellan gum ratio and medium pH, on nanoparticle characteristics, including size, zeta potential, encapsulation efficiency, drug loading degree and nanoparticle yield. Thus, the novelty of this work lies in the development of mucoadhesive polyelectrolyte nanoparticles based on chitosan and gellan gum as a potential carrier for pulmonary delivery of isoniazid, as well as in the optimization of the ethanol-based synthesis strategy and the systematic evaluation of formulation parameters that determine nanoparticle formation and drug encapsulation. Despite the growing interest in polymeric nanocarriers for pulmonary tuberculosis therapy, the potential of chitosan–gellan gum polyelectrolyte complex nanoparticles as carriers for isoniazid delivery remains largely unexplored. This study aims to address this gap by developing a mucoadhesive nanocarrier capable of providing prolonged pulmonary residence and controlled drug release.

## 2. Materials and Methods

### 2.1. Materials

Chitosan (low molecular weight, 50–190 kDa, ≥75% deacetylated), isoniazid (purity 99%) and fluorescein isothiocyanate (FITC) isomer I (≥90%; HPLC) were purchased from Sigma–Aldrich (Taufkirchen, Germany). Low-acyl gellan gum which used as a food additive, was used. Ethanol (90%, produced by Dosfarm, Almaty, Kazakhstan) was purchased from a pharmacy.

### 2.2. Preparation of Chitosan and Gellan Gum Stock Solutions

The CS solution (1 mg/mL) was prepared by the addition of chitosan powder in 0.1 M hydrochloric acid and stirred overnight at 25 °C. The GG solution (1 mg/mL) was prepared by stirring the gellan gum powder in deionized water at 80 °C for 1 h and then overnight at 60 °C. The gellan gum solution was filtered by cotton wool prior to use.

### 2.3. Preparation of Chitosan-Gellan Gum Nanoparticles (CSGG NPs)

The preparation of nanoparticles followed the method of chitosan-gellan gum gel preparation with modifications [13]. Different mass ratios of chitosan to gellan gum were mixed (total mass of polymers = 6 mg). For the nanoparticles prepared at pH 5, the pH values of both chitosan and gellan gum solutions were adjusted to pH 5 by adding 0.1 M of HCl solution or 0.2 M of NaOH solution (or more dilute HCl and NaOH solution if necessary) in advance. Gellan gum solution was added dropwise into chitosan solution at 60 °C. After stirring for 15 min, 10 mL of 90% ethanol was added dropwise and the reaction mixture was stirred for 2 h and the temperature was decreased from 60 °C to 25 °C simultaneously. Then the reaction mixture was taken for the measurements of particle size, polydispersity and zeta potential. The nanoparticles were then isolated by centrifugation at 15,060 rpm (Eppendorf 5420, Eppendorf SE, Hamburg, Germany) for 30 min and rinsed with distilled water by three cycles of centrifugation for 5 min each. The pellet was collected and dried. The optimal ratio of chitosan to gellan gum which produced CSGG NPs with the highest yield and the smallest size was determined.

### 2.4. Preparation of Chitosan-Gellan Gum Nanoparticles with Isoniazid (CSGG–INH NPs)

The optimal ratios of chitosan to gellan gum were chosen for loading the drug. For the nanoparticles prepared at pH 2 and pH 5, the pH values of both chitosan and gellan gum solutions were adjusted to pH 2 or 5 by adding 0.1 M of HCl solution or 0.2 M of NaOH solution (or more dilute HCl and NaOH solution if necessary) in advance. Then gellan gum solution with a fixed amount of isoniazid (6 mg) was added dropwise into chitosan solution at 60 °C. After stirring for 15 min, 10 mL of 90% ethanol was added dropwise and the reaction mixture was stirred for 2 h while the temperature was decreased from 60 °C to 25 °C simultaneously. Then the reaction mixture was taken for the measurements of particle size, polydispersity and zeta potential. The nanoparticles were isolated by centrifugation at 15,060 rpm (Eppendorf 5420, Hamburg, Germany) for 30 min to remove unencapsulated isoniazid and then rinsed with distilled water by three cycles of centrifugation for 5 min each. The pellet was collected and dried.

### 2.5. Preparation of FITC–Chitosan

The preparation procedure followed the methods reported by Cook et al. and Ge et al., with modifications [24,25]. To the chitosan solution (1% *w*/*v* in 0.1 M acetic acid; 1 mL), ethanol (1 mL) and FITC (2 mg/mL; 0.5 mL) were added, and the reaction mixture was stirred in the dark at 25 °C for 3 h, followed by precipitation in NaOH (0.1 M; 10 mL). FITC–chitosan was isolated by centrifugation at 15,060 rpm (Eppendorf 5420, Hamburg, Germany) for 30 min and washed with distilled water (15,060 rpm; 10 min each) until no fluorescence was detected in the supernatant using a fluorescence microscope (AOSVI, Shenzhen, China). The pellet was re-dissolved in 3 mL of 0.1 M acetic acid and dialyzed (MWCO: 8000–14,000 D, Viskase, Lombard, IL, USA) in 50 mL of distilled water for 3 days in the dark, with the water being replaced every day. The dialyzed product was dried by lyophilization.

### 2.6. Preparation of FITC–Chitosan–Gellan Gum Nanoparticles (FITC–CSGG NPs)

The preparation procedure was the same as described in Section 2.3, except that FITC–chitosan was used instead of chitosan. FITC–CSGG NPs were isolated by centrifugation at 15,060 rpm for 30 min and washed with distilled water (15,060 rpm; 10 min each) until no fluorescence was detected in the supernatant by using a fluorescence microscope. The pellet was collected and dried by lyophilization.

### 2.7. Preparation of FITC–Chitosan–Gellan Gum Nanoparticles with Isoniazid (FITC–CSGG–INH NPs)

The preparation procedure was the same as described in Section 2.4, except that FITC–chitosan was used instead of chitosan. FITC–CSGG–INH NPs were isolated by centrifugation at 15,060 rpm for 30 min and washed with distilled water (15,060 rpm; 10 min each) until no fluorescence was detected in the supernatant using a fluorescence microscope. The pellet was collected and dried by lyophilization.

### 2.8. Measurement of Particle Size, Polydispersity and Zeta Potential

The average diameter by intensity and polydispersity index (PDI) of nanoparticles were measured by Dynamic Light Scattering using Zetasizer Nano S90 device manufactured by Malvern Instruments Ltd. (Worcestershire, UK). Ten drops of nanoparticle suspension were added into 1.5–2 mL of distilled water in a quartz cuvette. The measurements were carried out at 25 °C at a scattering angle of 90°. The zeta potentials of nanoparticle suspensions were determined with a zeta-potential analyzer (NanoBrook ZetaPALS, Brookhaven, Holtsville, NY, USA) using electrophoretic laser Doppler anemometry.

### 2.9. Determination of the Yield of Nanoparticles, Drug Encapsulation Efficiency and Drug Loading Degree

The centrifugation of nanoparticle suspensions was carried out after the synthesis. The pellet was dried and the yield of nanoparticles was determined:

(1) $$Yield\;of\;NPs\left(\%\right)=\frac{mass\;of\;nanoparticles}{total\;mass\;of\;polymers+total\;mass\;of\;drugs}\times 100\%$$

In addition, the supernatant was collected to determine the amount of free drug by high performance liquid chromatography (HPLC) (Shimadzu LC-20 (Kyoto, Japan) Prominence equipment at 254 nm wavelength). Eluent (acetonitrile:water = 5:95) at a flow rate of 0.6 mL/min through a Promosil C18 column (CNW Technologies, Shanghai, China) (sorbent grain size 5 μm, 100 Å, 4.6 × 150 mm) was used for the separation of the components. The injection volume was 10 μL (loop injection).

The encapsulation efficiency and the drug loading degree were calculated according to the formulae below.

(2) $$Encapsulation\;efficiency\left(\%\right)=\frac{total\;mass\;of\;drug-mass\;of\;free\;drug}{total\;mass\;of\;drug}\times 100\%$$

(3) $$Loading\;degree\left(\%\right)=\frac{total\;mass\;of\;drug-mass\;of\;free\;drug}{mass\;of\;nanoparticles}\times 100\%$$

### 2.10. Morphological Characterization by Transmission Electron Microscopy and Nanoparticle Tracking Analysis

The nanoparticles were visualized using transmission electron microscopy (TEM; Talos L120C microscope, Thermo Fisher Scientific, Brno, Czech Republic). The samples for TEM were prepared by our fast-solvent-removal method combined with negative staining as described elsewhere [26]. Briefly, 3 μL of the nanoparticle solution was dropped onto a standard TEM grid covered with an electron-transparent carbon film, left to equilibrate for 2 min, and then the solution was removed by touching the bottom of the grid with a thin strip of filter paper. The dried samples were negatively stained with uranyl acetate solution, dried completely, and observed in the TEM microscope using bright field imaging at an accelerating voltage of 120 kV. The micrographs were processed with MyImg 0.6.0 [27], which is an open-source Python package (https://pypi.org/project/myimg accessed on 1 July 2026) for batch processing of light and electron micrographs. The nanoparticles were characterized by nanoparticle tracking analysis (NTA) using NanoSight NS300 (Malvern, Worcestershire, UK) [28]. For the NTA measurements, CSGG and CSGG−INH nanoparticle suspensions were diluted with deionized water. Fluorescence mode was used for the NTA measurement of the nanoparticles. 1 mL of diluted sample was loaded into the NTA syringe pump using a 1-mL syringe. A sCMOS camera and the NTA 3.4 software were used to capture and process the motion of the nanoparticles. The camera level was set to 10 and the detection thresholds was 4. A syringe pump speed of 25 AU was selected. The temperature was fixed at 25 °C.

### 2.11. Fourier-Transform Infrared Spectroscopy

The samples were analyzed by infrared spectroscopy with the FSM 1202 spectrometer from Infraspek Ltd. (Saint Petersburg, Russia). Fourier-transform infrared (FTIR) spectra were obtained using the KBr method. A pellet was prepared by blending around 3 mg of the sample with 100 mg of KBr. The scanning range was set from 4000 to 500 cm^−1^, with a resolution of 8 cm^−1^.

### 2.12. Thermogravimetric Analysis and Differential Scanning Calorimetry

Thermogravimetric and differential scanning calorimetry analyses were performed on a LabSYS evo TGA/DTA/DSC analyzer from Setaram (Setaram Instrumentation, Caluire-et-Cuire, France). The temperature spectrum was varied from 30 to 600 °C, employing an aluminum oxide crucible (Setaram Instrumentation, Caluire-et-Cuire, France). The sample was heated at 10 °C/min in a nitrogen atmosphere at a flow rate of 30.

### 2.13. In Vitro Release of Drug from Nanoparticles

The release of isoniazid from nanoparticles was studied by the dialysis method in a phosphate buffer solution (PBS, pH 7.4, 37 °C). In order to achieve this task, the CSGG–INH NPs were centrifuged and lyophilized. 90 mg of the dried CSGG–INH NPs were dispersed in 3 mL of PBS and treated with ultrasound for 10 min. The resulting dispersion was transferred into a dialysis membrane (MWCO: 1000 D). The membrane was closed on both sides with clip, and placed in a container with 50 mL of phosphate buffer in a thermostat at 37 °C. Aliquots (1 mL) of the release medium were collected at predetermined time points (5, 15 and 30 min; 1, 2, 4, 8 and 24 h). The amount of drug released from the polymer nanoparticles was determined by HPLC.

### 2.14. Mucoadhesion Study of Nanoparticles

Samples of FITC–chitosan, FITC–CSGG NPs and FITC–CSGG–INH NPs were separately applied to the surfaces of three ovine lung tissue samples [29]. The ovine lung tissue was cut into pieces of 2 × 2 cm^2^ and was mounted on a glass slide. The samples were mounted on microscope slides, inclined at 45°, and rinsed with phosphate buffer solution (pH 7.4) at 37 °C using a flow rate of 2 mL/min until a total volume of 500 mL was delivered. Each experiment was conducted for more than 4 h. After every 100 mL of buffer had been applied, the amounts of the samples remaining on the lung tissue were determined by fluorescence microscopy under blue light with the software ImageJ 1.54 m. The results were expressed as normalized intensity.

### 2.15. Determination of Antimycobacterial Activity of CSGG–INH Nanoparticles

In vitro studies were performed in Safemate Ecoplus 1,5. (Bioair, Lombardy, Italy) Biosafety cabinets which provided sterile conditions through negative pressure and air filtration through HEPA filters. The antimycobacterial activity of the nanoparticles was evaluated using an antituberculosis-sensitive wild strain of MTB H37Rv, obtained from Asklepios Pulmonology Clinic (Munich-Hauting, Germany). Sterile Mycobacteria Growth Indicator Tube (MGIT) tubes were pre-filled with 800 µL of a nutrient supplement (MGIT OADC supplement) containing the components necessary to support the growth and viability of MTB, including nutrients, vitamins, and growth factors.

In order to investigate antimycobacterial activity, chitosan-gellan gum nanoparticles were synthesized without drug and also loaded with isoniazid under optimized conditions. CSGG–INH NPs were weighed so that the amount of isoniazid in each sample was 10, 20, 40, 60, and 80 mg. After adding the nanoparticles to the MGIT liquid system, prolonged incubation was carried out for 25 days. To confirm the presence or absence of viable MTB cells, control subcultures were performed from the MGIT liquid medium onto solid Löwenstein–Jensen medium. For each experimental group, material from the MGIT tubes was transferred to the surface of the Löwenstein–Jensen medium, followed by incubation at 36 ± 1 °C. The tubes containing the cultures were incubated for 4 weeks, after which the results were recorded. The results of suppressive activity were evaluated by counting the number of colony-forming units (CFU) of MTB on the nutrient medium and comparing the control with the experimental cultures. For samples showing positive results, smears were made and stained according to the Ziehl–Neelsen method [30]. The smear was allowed to air dry and examined microscopically using a Leica DMLS (Leica Microsystems GmbH, Wetzlar, Germany) binocular microscope.

### 2.16. In Vitro Toxicity Evaluation Using Artemia Salina Nauplii

The toxic activity of the produced nanoparticles was evaluated using a survival test with *Artemia salina* nauplii. Artemia salina eggs were incubated in artificial seawater under aeration at 25 °C for 48–72 h until hatching. After hatching, 20–40 nauplii in 990 µL of seawater were placed in a 2 mL well of a laboratory plate, after which 10 µL of extract solution at the appropriate concentration was added. The number of dead individuals was counted after 24 h. The mortality percentage (P) was calculated using the following formula:

$$P=\frac{A-N}{Z}\times 100\%$$

where A is the number of dead larvae in the test, N is the number of dead larvae before the start of the experiment, and Z is the total number of larvae in the well at the start of the experiment. The experiment was performed in three independent replicates. The concentrations tested were 0.1, 0.5, and 1 mg/mL. The LC_50_ value was calculated by probit regression.

### 2.17. Statistical Processing of the Obtained Data

All studies were performed at least three times. The results are presented as the mean with standard deviation. One-factor analysis of variance (ANOVA) followed by Tukey’s test was used to compare independent groups. The analysis was performed using Minitab 19 software, and the level of significance was set at *p* < 0.05.

## 3. Results

### 3.1. Synthesis and Characterization of Chitosan-Gellan Gum Nanoparticles

Polyelectrolyte complexes are formed as a result of electrostatic interactions between chitosan polycations and gellan gum polyanions. The driving force behind the formation of PECs is the increase in entropy, which occurs due to the release of counterions during the process. Consequently, the characteristics and efficiency of PEC formation are significantly affected by the number of charges present on the polymer chains, which affects the electrostatic attraction between the components [11,12]. There are various methods for the preparation of polyelectrolyte complexes, which provide certain advantages depending on the desired application. One of the methods is ionotropic gelation, which involves spontaneous attraction between polyelectrolyte and counterions [31]. Polyelectrolyte complexation is the simplest method in which oppositely charged polyelectrolytes form nanoparticles during electrostatic interaction [32,33]. Another technique, known as complex coacervation, leads to liquid-liquid phase separation to form a polymer-rich dense phase and a polymer-poor dilute supernatant [34]. Flash nanoprecipitation provides homogeneous mixing complexation between polyelectrolytes to form nanocomplexes with a narrow size distribution and controllable size [35,36]. Ultra-high pressure homogenization is a top-down method, which is based on a high-energy dispersion process to disaggregate a coarse system to form nanoparticles [33].

**Figure 1:**
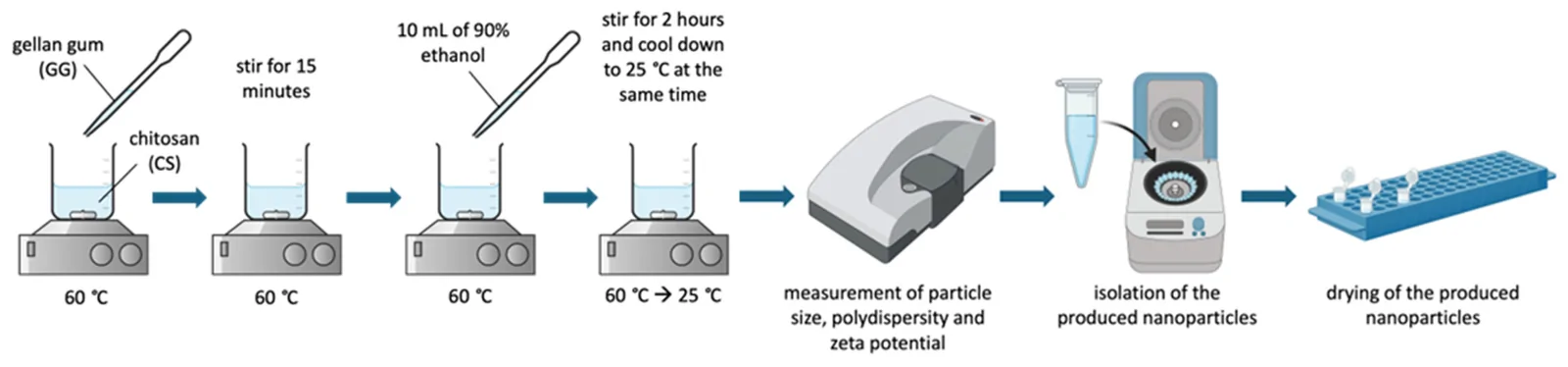
Schematic representation of the CSGG NPs formation process.

In this work, the polyelectrolyte complex coacervation method was used to produce CSGG NPs as it is the simplest method to form colloidal PECs without the need for special equipment. In contrast to the ordinary polyelectrolyte complex coacervation method for the synthesis of PEC nanoparticles, in our method, ethanol was added in order to produce nanoparticles with better physicochemical properties [37]. Schematic representation of the nanoparticle formation process is shown in Figure 1.

**Figure 2:**
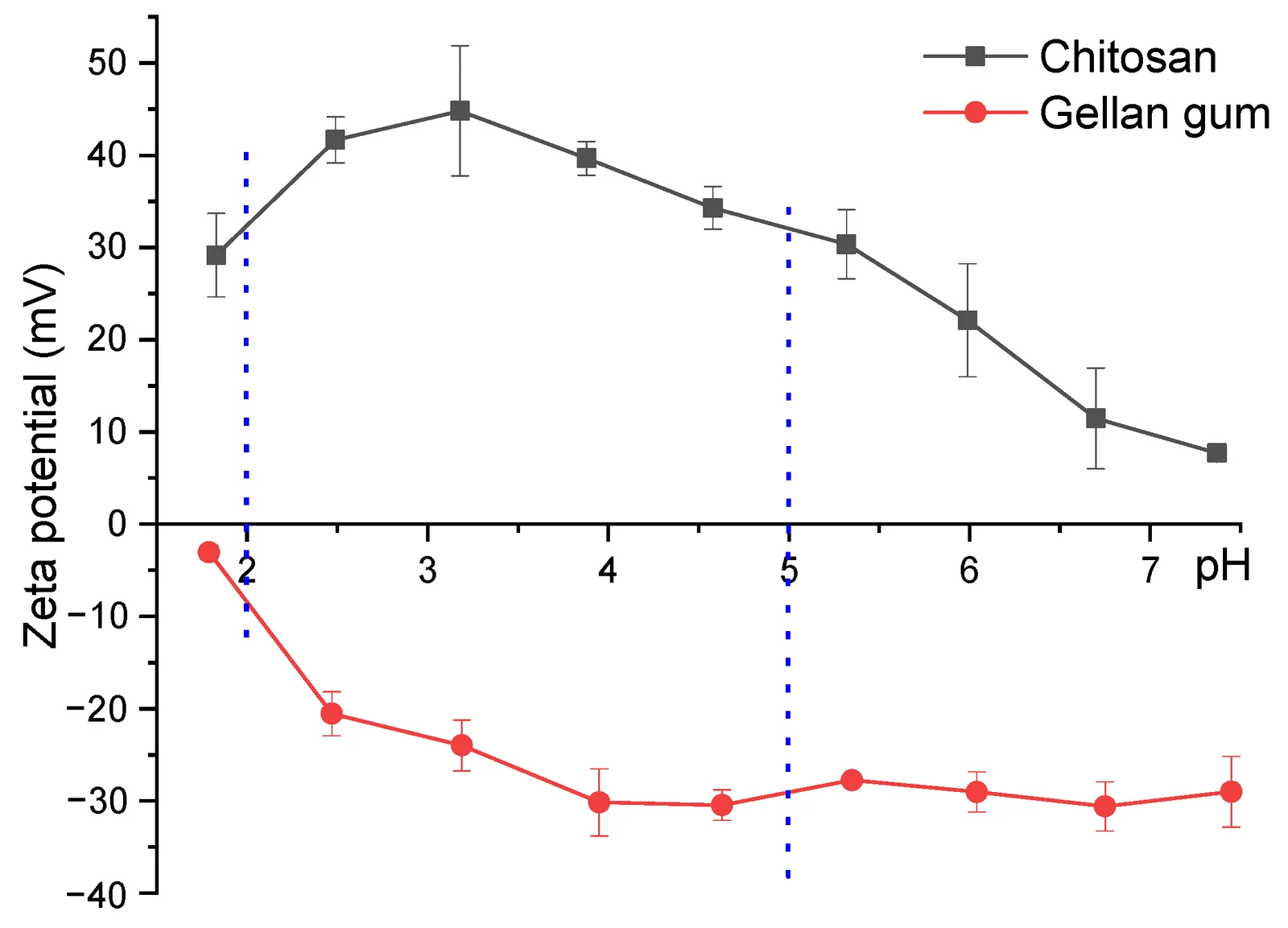
Effect of pH on the zeta potential of chitosan and gellan gum dispersions. Data are expressed as mean ± standard deviation (n = 3). Blue dashed lines indicate the pH values (2 and 5) selected for subsequent nanoparticle preparation based on the zeta-potential profiles.

Since chitosan contains positively charged amino groups and gellan gum contains negatively charged carboxyl groups, it can be assumed that changing the synthesis conditions will affect the characteristics of the final product. There are several parameters that can be optimized in the synthesis, including solution pH and the chitosan-to-gellan gum ratio. Among these, pH plays a particularly important role because it determines the ionization state of both polymers and, consequently, the strength of the electrostatic interactions responsible for nanoparticle formation [7]. Therefore, the surface charge of the individual polymer dispersions was first investigated over the selected pH range. The zeta potentials of dispersions of chitosan and gellan gum at different pH values are shown in Figure 2. The zeta potential of chitosan was positive while the zeta potential of gellan gum was negative in the pH range studied, which was related to the protonation of its amino groups and the dissociation of its carboxyl groups, respectively. With the rising pH, the positive charge density of chitosan gradually decreased, while the negative charge density of gellan gum increased. Chitosan has a high positive charge density at pH 2, while gellan gum carries only a small number of negative charges since most of its carboxyl groups are protonated. At pH 5, however, both polymers were highly ionized, leading to a more balanced distribution of charges and higher electrostatic interactions [38]. Considering these results, two pH values (pH 2 and 5) were chosen for the preparation of nanoparticles.

The influence of the chitosan-to-gellan gum mass ratio and pH on the physicochemical characteristics of CSGG nanoparticles is presented in Table 1. Nanoparticles formed successfully across a wide range of polymer ratios at both pH 2 and pH 5, indicating that chitosan and gellan gum can form stable polyelectrolyte complexes through electrostatic interactions.

By varying the ratio of chitosan to gellan gum (from 9:1 to 1:9), nanoparticles with different sizes, PDI values, and zeta potentials were obtained. At pH 2, the particle sizes ranged from 214 ± 15 nm to 358 ± 6 nm, the PDI values ranged from 0.042 ± 0.020 to 0.431 ± 0.092, and the nanoparticle yields ranged from 27% to 63%. The zeta-potential values were mostly positive (range 17 ± 2 mV to 28 ± 2 mV), indicating that the nanoparticles may contain more protonated amino groups of chitosan. Of all formulations, only the formulation with the highest gellan gum content (C1G9) had a negative zeta potential (−10 ± 2 mV), indicating that gellan gum chains had accumulated on the nanoparticle surface. The nanoparticle yield generally increased with increasing gellan gum content, reaching 63% for C4G6, indicating more efficient particle formation when sufficient anionic polymer was available for complexation.

At pH 5, the characteristics of the nanoparticles showed a greater dependence on polymer composition. Formulations with high chitosan content (C9G1-pH5 and C8G2-pH5) gave relatively large particle sizes (522 ± 16 nm and 619 ± 16 nm, respectively) with high PDI values (>0.59), indicating aggregation and a decrease in colloidal stability. The smallest nanoparticles were obtained for C1G9-pH5, with an average diameter of 124 ± 1 nm and a PDI of 0.252 ± 0.020. A gradual decrease in zeta potential was also observed with increasing gellan gum content, shifting from strongly positive values for C8G2-pH5 (29 ± 2 mV) to negative values for gellan gum-rich formulations C2G8-pH5 (−26 ± 2 mV). This shift indicates an increasing number of deprotonated carboxyl groups of gellan gum on the nanoparticle surface.

After determining the physicochemical characteristics, the optimal compositions at each pH were selected for further isoniazid loading experiments. Composition C4G6, which had the smallest particle size (214 ± 15 nm), a relatively narrow size distribution (PDI = 0.270 ± 0.012), a high positive zeta potential (28 ± 2 mV), and a satisfactory nanoparticle yield (63%), was selected from among the nanoparticles obtained at pH 2. These characteristics and its good colloidal stability suggested that C4G6 was a promising carrier for drug encapsulation. The C2G8-pH5 formulation was selected for the nanoparticles obtained at pH 5 due to its particle size (221 ± 10 nm), low polydispersity index (0.134 ± 0.038), high nanoparticle yield (73%), and high zeta potential (−26 ± 2 mV), which indicates its good colloidal stability. The C1G9-pH5 formulation resulted in a smaller particle size but a much lower yield, whereas the surface charge of C2G8-pH5 was potentially advantageous for further drug loading experiments. Thus, the C4G6 and C2G8-pH5 formulations were selected for isoniazid loading, and the results are presented in Table 2.

The loading of isoniazid changed the size and zeta potentials of the NPs. The diameters of C4G6-INH and C2G8R-INH-pH5 were 178 ± 7 nm and 185 ± 3 nm, respectively. The addition of isoniazid altered chitosan-gellan gum interactions and introduced new polymer-drug interactions, thereby affecting nanoparticle size [39]. The zeta potential remained positive for C4G6-INH (+22 ± 3 mV), indicating that protonated amino groups of chitosan were still predominant on the nanoparticle surface after drug encapsulation. In contrast, C2G8-INH-pH5 exhibited a negative zeta potential (−15 ± 1 mV), reflecting the higher proportion of gellan gum and the presence of exposed carboxyl groups. The decrease in the zeta potential after drug loading suggested partial interaction of isoniazid molecules with charged functional groups of the polymers, which reduced the net surface charge of the nanoparticles [40].

There was a significant difference in the encapsulation efficiency of the two formulations. C4G6-INH exhibited an encapsulation efficiency of 32% and a loading degree of 61%, whereas C2G8-INH-pH5 showed significantly lower values of 18% and 22%, respectively. The higher encapsulation efficiency observed for C4G6-INH may be related to its higher chitosan content and the more favorable CS/GG ratio for nanoparticle formation, which may promote the formation of a denser polyelectrolyte complex network capable of retaining isoniazid within the nanoparticle matrix. Despite C2G8-INH-pH5 showing a higher nanoparticle yield (42%) than C4G6-INH (26%), its significantly lower encapsulation efficiency and drug loading suggest a reduced ability to retain isoniazid within the polymer matrix. Therefore, from a drug delivery perspective, the C4G6 formulation prepared at pH 2 exhibited superior characteristics and was chosen for further investigations. These results suggest that acidic preparation conditions and a balanced chitosan-to-gellan gum ratio contribute to the formation of nanoparticles with enhanced isoniazid encapsulation capacity.

### 3.2. Study of the Physicochemical Properties of the Produced CSGG–INH Nanoparticles

**Figure 3:**
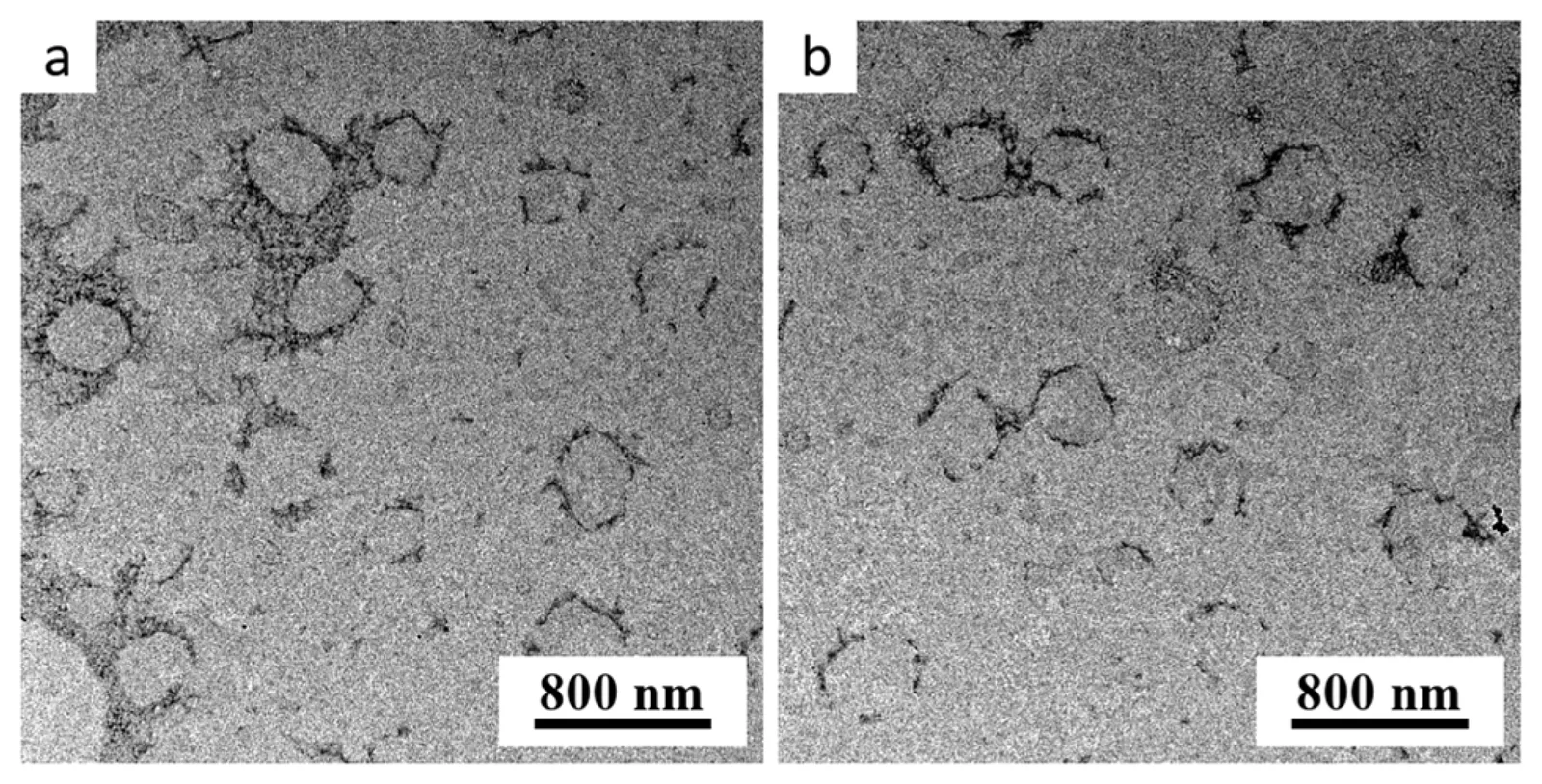
TEM micrographs of chitosan-gellan gum NPs: (**a**) selected region showing negatively stained spherical particles; (**b**) another region showing similar particles, confirming the reproducibility of the TEM observations.

Nanoparticles of the polyelectrolyte complex without drug (CSGG NPs) and loaded with isoniazid (CSGG–INH NPs) were further analyzed using transmission electron microscopy (TEM) and nanoparticle tracking analysis (NTA). TEM micrographs (Figure 3) showed the formation of spherical nanosized structures with reasonably narrow size distributions. The average particle size estimated from TEM micrographs (306 ± 69 nm) was higher than that measured by DLS (214 ± 15 nm). This discrepancy is likely due to particle deformation during TEM sample preparation, which comprised drying and negative staining. These processescould lead to particle flattening on the carbon film and an increase in the apparent particle diameter [41,42].

In addition, NTA was used to further characterize the nanoparticles by tracking their Brownian motion. The particle size measured by NTA for CSGG NPs was 215 ± 17 nm and for CSGG–INH NPs was 148 ± 17 nm. The NTA measurements were in the same nanoscale range with the particle size values obtained by intensity DLS, confirming the reliability of the results. Furthermore, the recorded video (Videos S1 and S2) showed that the nanoparticles were predominantly spherical in shape and were well dispersed in the suspension without significant aggregation.

**Figure 4:**
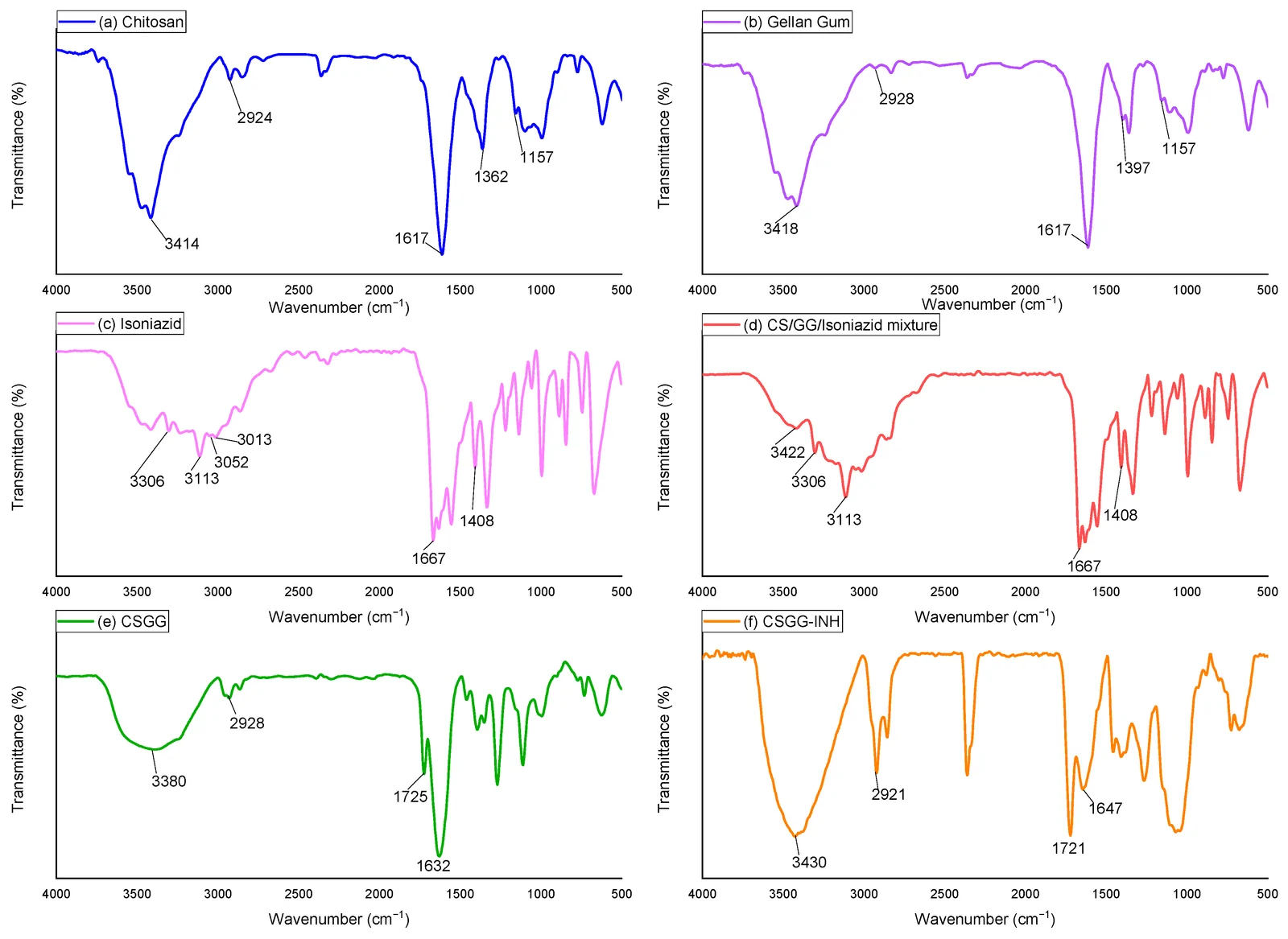
Fourier-transform infrared spectra of (**a**) chitosan, (**b**) gellan gum, (**c**) isoniazid, (**d**) chitosan/gellan gum/isoniazid mixture, (**e**) CSGG NPs and (**f**) CSGG–INH NPs.

Fourier-transform infrared (FTIR) spectroscopy was performed to confirm the interaction between the polyelectrolyte complex components and isoniazid encapsulation. Figure 4 shows the infrared spectra of chitosan, gellan gum, isoniazid, chitosan/gellan gum/isoniazid mixture, nanoparticles without isoniazid (CSGG) and with isoniazid (CSGG–INH).

In Figure 4a, several characteristic peaks of chitosan were observed, including the peaks at 3414 cm^−1^ (O–H stretching (str.) and N–H str.), 2924 cm^−1^ (C–H str.), 1617 (C=O in NHCOCH_3_), 1362 cm^−1^ (CH_3_ in NHCOCH_3_) and 1157 cm^−1^ (C–O–C) [43]. The gellan gum spectrum (Figure 4b) showed peaks at 3418 cm^−1^ (O–H str.), 2928 cm^−1^ (C–H str.), 1617 cm^−1^ (asymmetric COO^−^ stretching (asy. str.)), 1397 cm^−1^ (symmetric COO^−^ stretching (sym. str.)) and 1157 cm^−1^ (C–O str.) [44,45]. In the spectrum of isoniazid (Figure 4c), the peaks were exhibited at 3306 cm^−1^ (N–H str.), 3113 cm^−1^ (sym. N_2_H_2_ str.), 3052 cm^−1^ (asy. N_2_H_2_ str.), 3013 cm^−1^ (C–H str.), 1667 cm^−1^ (C=O) and 1408 cm^−1^ (ring str.) [46,47]. For chitosan/gellan gum/isoniazid mixture (Figure 4d), the peaks observed in chitosan, gellan gum and isoniazid were found, including the peaks at 3422 cm^−1^ (O–H str. and N–H str. in chitosan, and O–H str. in gellan gum), 3306 cm^−1^ (N–H str. in isoniazid), 3113 cm^−1^ (sym. N_2_H_2_ str. in isoniazid), 1667 cm^−1^ (C=O in isoniazid) and 1408 cm^−1^ (ring str. in isoniazid). In the spectrum of PEC without isoniazid (CSGG) (Figure 4e), the peak at 2928 cm^−1^ corresponding to the stretching of C–H bonds was observed. The peak at 3380 cm^−1^ which is attributed to the O–H stretching and N–H stretching of chitosan and the O–H stretching of gellan gum, was found. It showed a small shift when compared with the corresponding peaks observed in Figure 4a and Figure 4b. Moreover, a new peak was found at 1725 cm^−1^ (C=O), and a peak with a displacement at 1632 cm^−1^ (C=O) was observed. As this result was not exhibited in the chitosan/gellan gum/isoniazid mixture (Figure 4d), it is probably due to the supramolecular interactions between chitosan and gellan gum in the PEC. It might be attributed to the interactions between the N–H group and the C=O group, and between the O–H group and the C=O group in the PEC. For the spectrum of PEC loaded with isoniazid (CSGG–INH) (Figure 4f), the peak at 2921 cm^−1^ (C–H str.) was observed. The peak at 1721 cm^−1^ (C=O) was found, which is probably due to the supramolecular interactions between chitosan and gellan gum in the PEC. When compared with Figure 4e, the peaks at 3430 cm^−1^ (O–H str. and N–H str. in the PEC) and 1647 cm^−1^ (C=O) showed small shifts. These changes in the FTIR spectra suggested the presence of intermolecular interactions between isoniazid and the chitosan–gellan gum polyelectrolyte complex [48,49].

**Figure 5:**
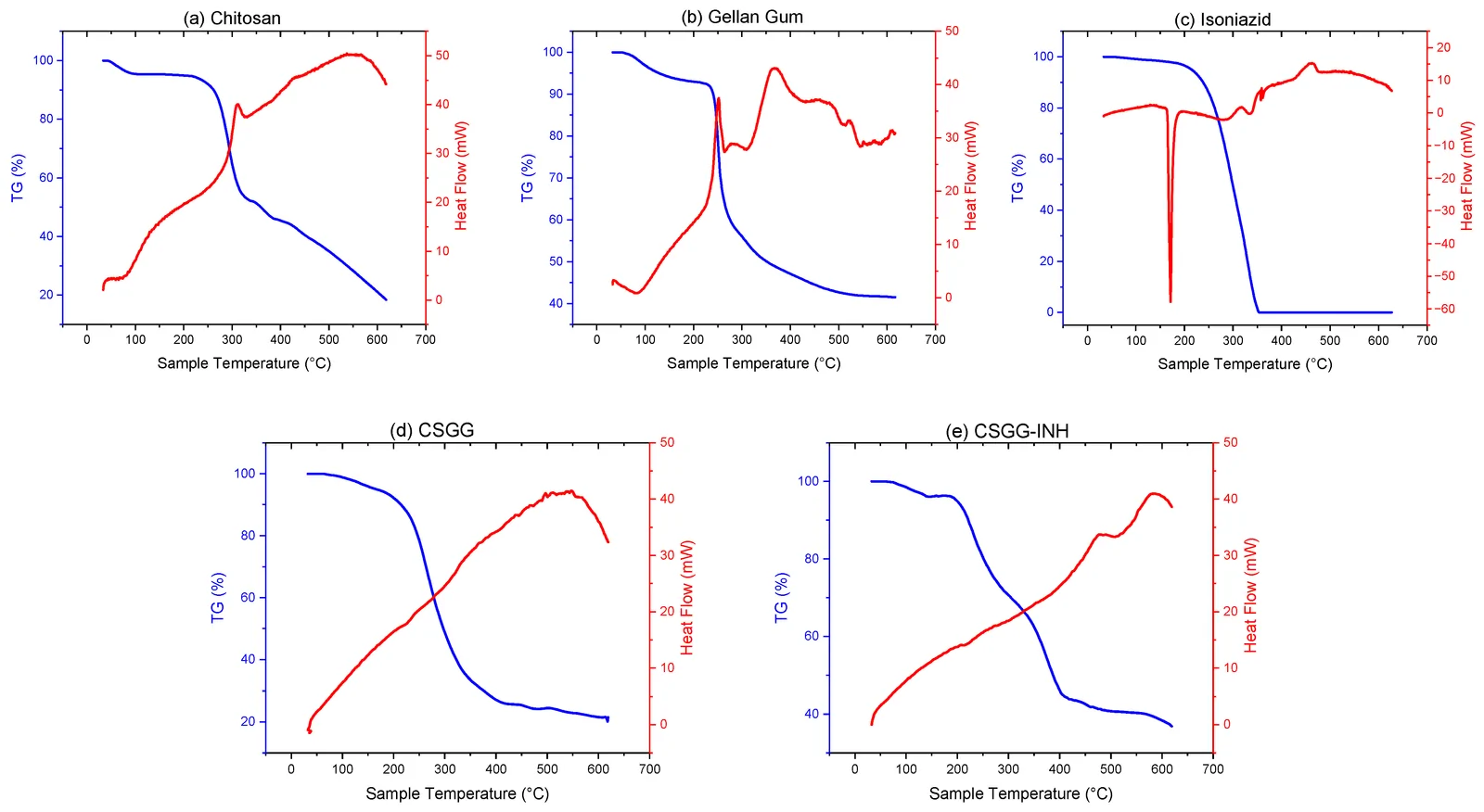
TGA and DSC of (**a**) chitosan, (**b**) gellan gum, (**c**) isoniazid, (**d**) CSGG NPs, and (**e**) CSGG–INH NPs.

Thermogravimetric analysis (TGA) and differential scanning calorimetry (DSC) provide the information of mass loss and heat flow on the samples while increasing temperature (Figure 5).

Figure 5a shows an exothermic peak at 309 °C with mass loss, and it is related to the decomposition of the amine units in chitosan [50]. In Figure 5b, an exothermic peak at 252 °C with mass loss was observed, which is probably due to the decomposition of gellan gum. For isoniazid (Figure 5c), an endothermic peak at 171 °C was found without mass loss. Thisshowed that isoniazid is crystalline and it melts at this temperature [51]. In Figure 5d, the exothermic peaks of both chitosan and gellan gum were not observed in chitosan-gellan gum PEC. There is no melting peak of isoniazid in Figure 5e, indicating that isoniazid is amorphous and molecularly dispersed in the nanosized polyelectrolyte complex [52]. The TGA results indicated that CSGG–INH nanoparticles exhibited improved thermal stability compared with the unloaded chitosan–gellan gum nanoparticles. This behavior may be attributed to the successful incorporation of isoniazid within the polymeric matrix, which could enhance intermolecular interactions and result in a more compact and thermally stable polyelectrolyte network [53].

### 3.3. Mucoadhesion Study of PEC Nanoparticles on Ovine Lung Tissue

**Figure 6:**
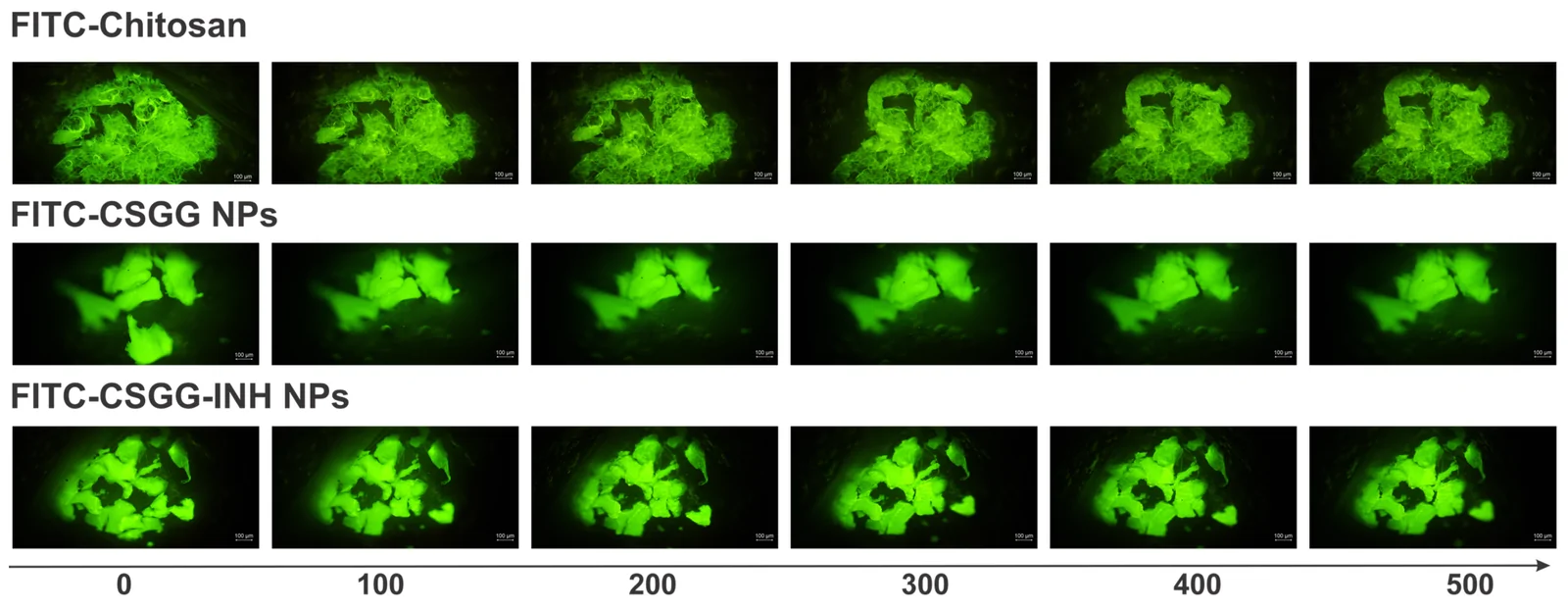
Fluorescence images showing the mucosal retention of FITC–chitosan, FITC–CSGG NPs and FITC–CSGG–INH NPs on ovine lung tissue after washing with varying volumes of PBS (pH 7.4; flow rate 2 mL/min). Scale bars correspond to 100 µm.

To determine the mucoadhesive properties of the obtained PEC nanoparticles, we used a modified fluorescence retention assay [26]. For this purpose, samples labeled with fluorescein isothiocyanate (FITC) were synthesized: chitosan (FITC–Chitosan), chitosan-gellan gum nanoparticles (FITC–CSGG NPs), and chitosan-gellan gum-isoniazid nanoparticles (FITC–CSGG–INH NPs). Fresh ovine lung tissue was continuously washed with phosphate buffer solution (PBS, pH 7.4) at 37 °C at a flow rate of 2 mL/min. The retention profiles of fluorescently labeled chitosan and nanoparticles were evaluated using fluorescence microscopy (Figure 6).

The images showed that all polymer composites remained on the lung tissue throughout the washing experiments, confirming their good mucoadhesive properties. FITC–chitosan exhibited excellent fluorescence across the entire tissue surface after washing with 500 mL of PBS, as chitosan is known to bind to mucin via electrostatic interactions between the protonated amino groups in chitosan and the negative charges of the sialic acid residues in mucin glycoproteins [54]. FITC–CSGG NPs also exhibited consistent fluorescence, indicating that the presence of gellan gum did not affect the adhesive properties of chitosan. Notably, FITC–CSGG–INH NPs also retained high fluorescence intensity after prolonged washing, indicating that the encapsulation of isoniazid does not adversely affect the adhesion of the nanoparticles to the lung tissue.

**Figure 7:**
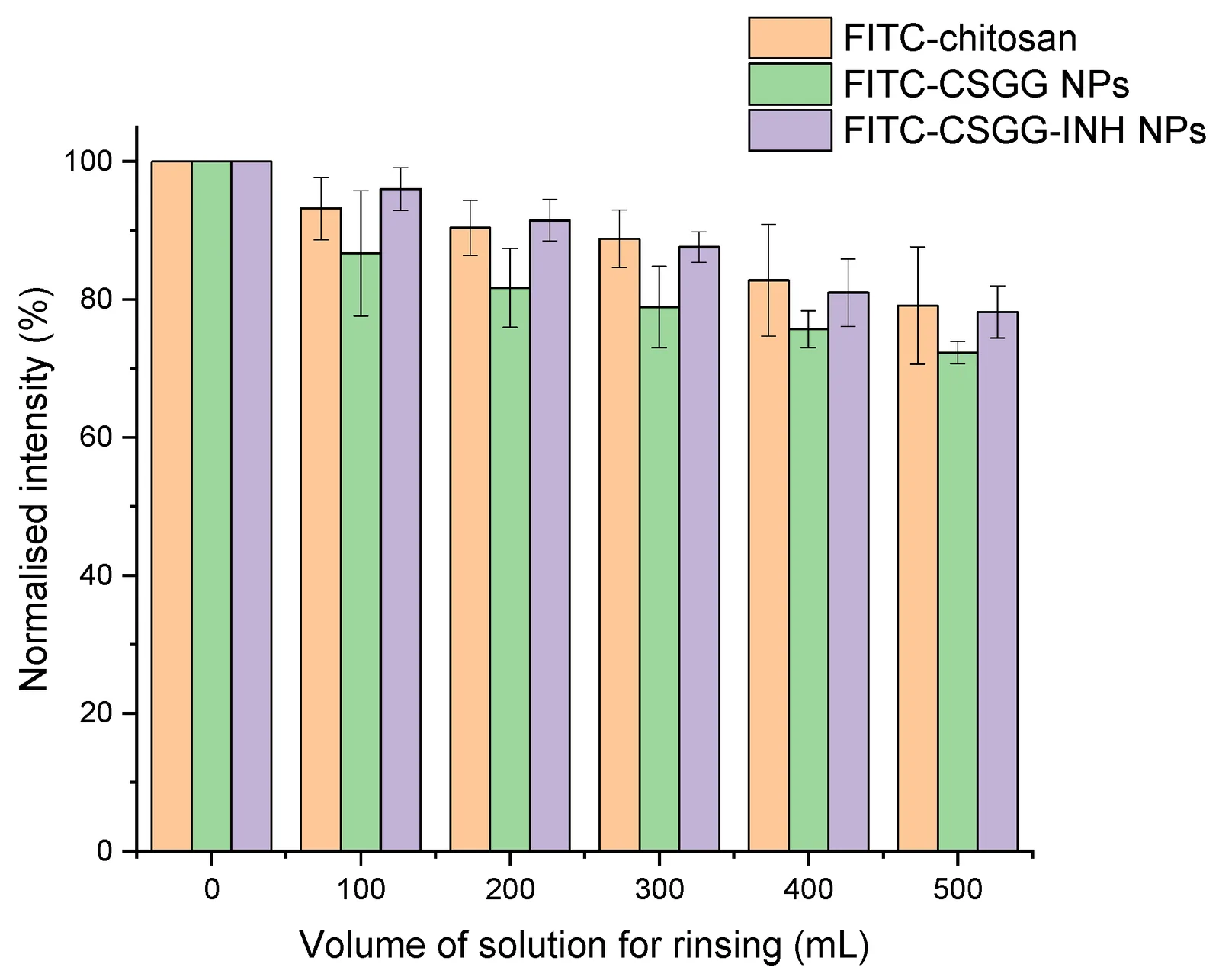
Percentage retention of FITC–chitosan, FITC–CSGG NPs, and FITC–CSGG–INH NPs on ovine lung tissue after washing with varying volumes of PBS (pH 7.4; flow rate 2 mL/min). Data are expressed as mean ± standard deviation (n = 3).

The quantitative analysis of the fluorescent micrographs using ImageJ showed that the fluorescence decreased gradually as the volume of the wash increased for all the formulations, indicating that the weakly adhered material was gradually removed from the surface of the lung tissue (Figure 7). However, a significant portion of the fluorescent signal remained after washing with 500 mL of PBS, confirming the strong mucoadhesive properties of the polymer systems. For all wash volumes, no statistically significant differences (*p* > 0.05) were found between FITC–chitosan, FITC–CSGG NPs, and FITC–CSGG–INH NPs. These results indicated that the inclusion of gellan gum and the encapsulation of isoniazid do not adversely affect the intrinsic mucoadhesive properties of chitosan. Preserved mucoadhesion is probably attributed to the presence of protonated amino groups on the nanoparticle surface that can still interact with the negatively charged mucin, even after the chitosan-gellan polyelectrolyte complex is formed.

### 3.4. Study of Isoniazid Release from the Polymer Matrix In Vitro

**Figure 8:**
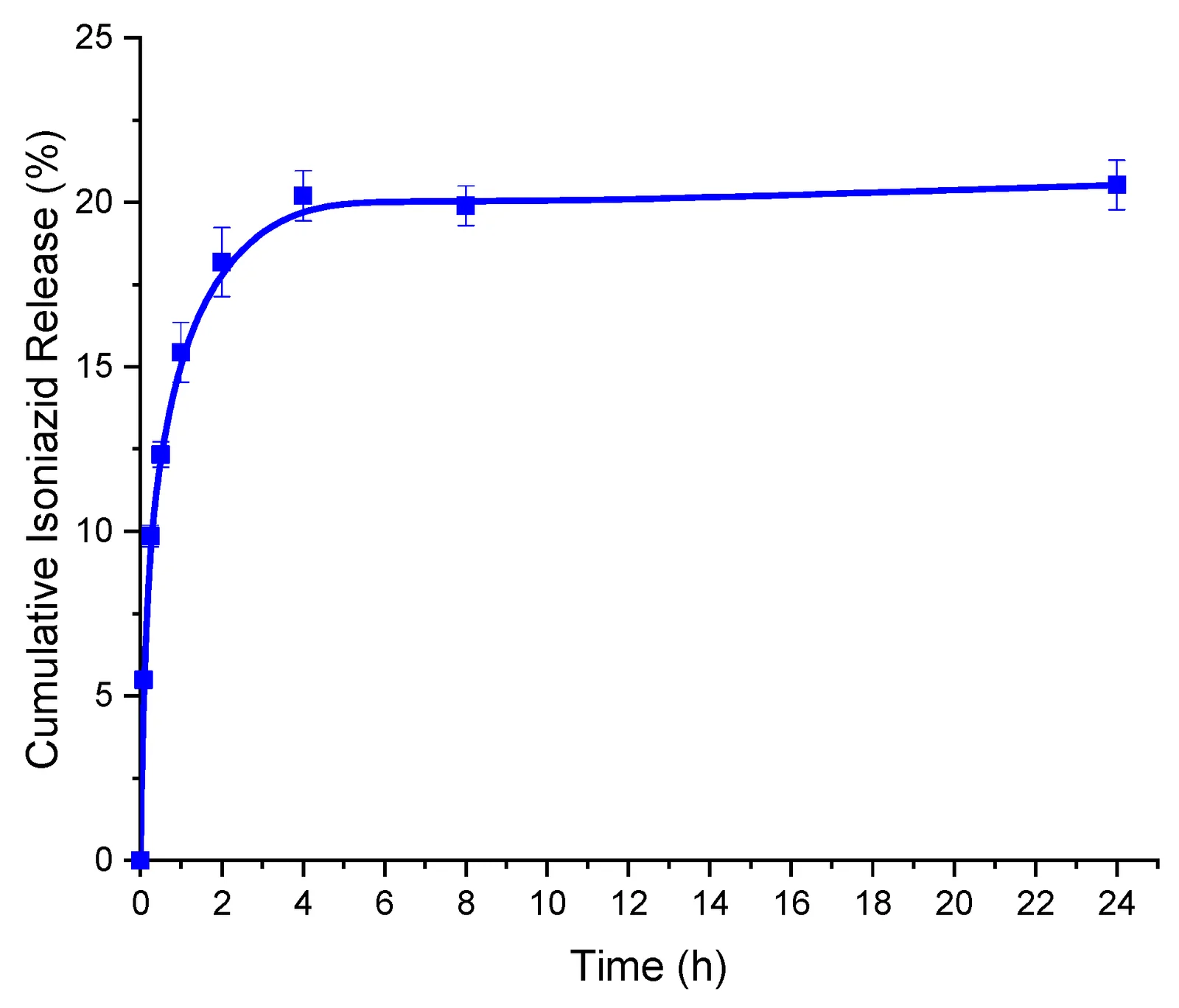
Cumulative isoniazid release from CSGG–INH NPs by the dialysis method in a phosphate buffer solution at pH 7.4. Data are expressed as mean ± standard deviation (n = 3).

To investigate the degree of isoniazid release from a chitosan-gellan gum polymer matrix, the dialysis method was employed in a phosphate buffer solution at pH 7.4, under physiological pH conditions (Figure 8).

The drug release exhibited a biphasic behavior, with an initial ‘burst’ followed by a prolonged release phase. The initial ‘burst’ of release is associated with the rapid diffusion of isoniazid adsorbed onto the surface of the nanoparticles [55,56]. Following this stage, drug release becomes controlled, and isoniazid is gradually released from the polymer matrix as the polymer swells or degrades.

**Figure 9:**
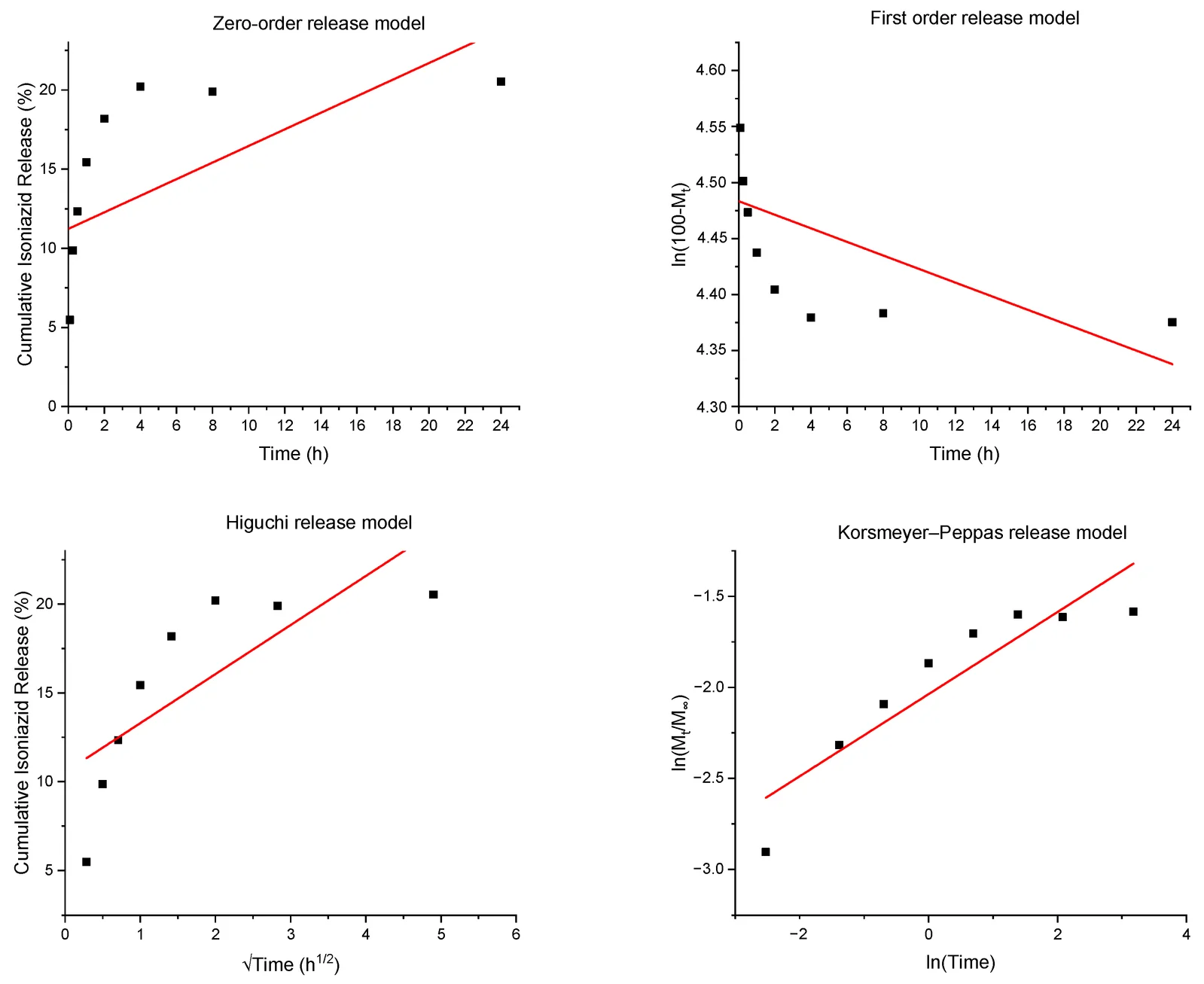
Kinetic data were fit to different models, reflecting isoniazid release from the CSGG nanoparticles in solution at pH 7.4.

After 24 h, more than 20% of the isoniazid had been released; thus, the nanoparticles developed are capable of sustaining prolonged release under physiological conditions. For the delivery of a pulmonary drug, such a release profile may be advantageous, as it could contribute to sustained local drug availability. Furthermore, the prolonged release behaviour, combined with the pronounced mucoadhesive properties demonstrated in this study, supports the further investigation of CSGG–INH nanoparticles as potential carriers for pulmonary delivery of anti-tuberculosis drugs. Four mathematical models were used to evaluate the drug release mechanism from nanoparticles: zero-order, first-order, Higuchi, and Korsmeyer–Peppas (Figure 9) [57,58,59]. Calculations were performed based on the average values of three parallel measurements of cumulative drug release. The resulting relationships were evaluated using the coefficient of determination R^2^, which characterizes the degree of agreement between the experimental data and the corresponding kinetic model.

The zero-order model showed a low correlation with the experimental data (R^2^ = 0.349). The linear equation was M_t_ = 0.403t + 13.235, indicating that the release rate was not constant over the study period. This is consistent with the experimental graph (Figure 8), which showed a rapid increase in the amount of released drug during the initial stages, followed by a prolonged release. For the first-order model, the determination coefficient was R^2^ = 0.360 (Equation: ln(100 − M_t_) = −0.00475t + 4.462), which also indicates that the model does not adequately fit the experimental data. The Higuchi model demonstrated a better fit to the data compared to the previous models. For this model, R^2^ = 0.588 was obtained, and the linear relationship equation was M_t_ = 2.763√t + 10.534. A higher R^2^ indicates a certain contribution of the diffusion mechanism to the release from nanoparticles. The best fit to the experimental data was obtained using the Korsmeyer–Peppas model (R^2^ = 0.836). The linearized equation is ln(M_t_/M_∞_) = 0.226 ln(t) − 2.037, where the release exponent n = 0.226 and K = 0.130. The higher R^2^ value compared to the other models studied indicates that the Korsmeyer–Peppas model most accurately describes the experimental release profile. The obtained value of n = 0.226 indicates that drug transport is predominantly diffusive in nature [60,61].

Taken together with the results of the Higuchi model, these findings suggest that the diffusion of INH from the polymer matrix of the nanoparticles is one of the main mechanisms governing its release. Thus, in the initial stage, the drug adsorbed onto the particle surface is released, followed by a slower release of the drug from the interior of the polymer matrix. These results indicate that the release of INH from nanoparticles is predominantly diffusion-controlled rather than governed constant-rate kinetics.

### 3.5. Investigation of the Antimycobacterial Activity of CSGG–INH NPs

**Figure 10:**
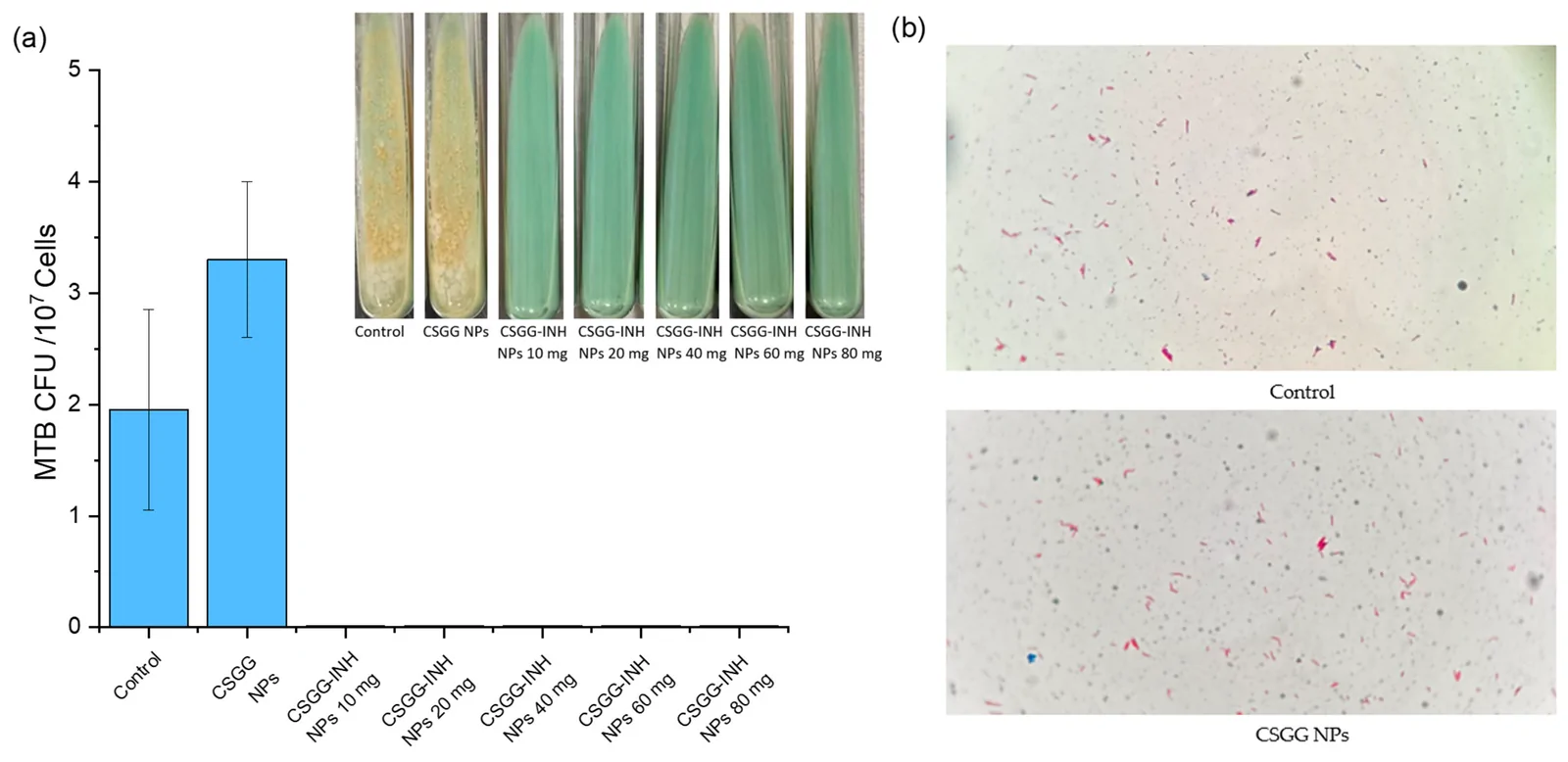
(**a**) Results of the antimycobacterial activity test of the CSGG and CSGG–INH nanoparticles on the growth of H37Rv on Löwenstein–Jensen nutrient-dense medium. Data are expressed as mean ± standard deviation (n = 3). (**b**) Microscopic analysis of smears from MTB H37RV strains on specimens stained using the Ziehl–Neelsen method.

The antimycobacterial activity of the developed chitosan-gellan nanoparticles loaded with isoniazid (CSGG–INH NP) was tested against MTB H37Rv using the Mycobacteria Growth Indicator Tube (MGIT) liquid culture system. The results are shown in Figure 10. As expected, abundant growth of MTB was observed in the control groups (i.e., the control without nanoparticles and the chitosan-gellan nanoparticles without drug) following incubation and subsequent subculturing on Löwenstein–Jensen medium, confirming the viability of the bacterial culture and the suitability of the experimental model. Microscopic examination of smears stained using the Ziehl–Neelsen method further confirmed the presence of numerous acid-fast bacilli in the control groups (Figure 10b).

In contrast, no visible growth of MTB H37Rv was detected on Löwenstein–Jensen medium following exposure to CSGG–INH nanoparticles at any tested concentration (10, 20, 40, 60, and 80 mg). The absence of bacterial growth following transfer from the MGIT system indicates that the nanoparticles released a sufficient quantity of isoniazid during the incubation period to completely inhibit the drug-sensitive strain. The prolonged incubation period in the MGIT medium also indicates the sustained-release behaviour of the developed nanoparticles. The lyophilized chitosan-gellan nanoparticles slowly hydrate and swell during incubation, allowing isoniazid to be released into the incubation medium. Consequently, therapeutic levels were maintained throughout the incubation period, and all bacterial growth was suppressed.

These results are consistent with in vitro drug release studies, which demonstrated sustained release of isoniazid from the chitosan-gellan matrix. The combination of controlled drug release and complete inhibition of H37Rv MTB highlights the potential of CSGG–INH nanoparticles as a nanocarrier for isoniazid delivery and supports their further investigation for pulmonary drug delivery applications. In addition to their high mucoadhesive properties, the produced nanoparticles may also increase the residence time in the lungs and enhance the efficacy of localized tuberculosis treatment.

### 3.6. Toxicity of Isoniazid and CSGG Nanoparticles

**Figure 11:**
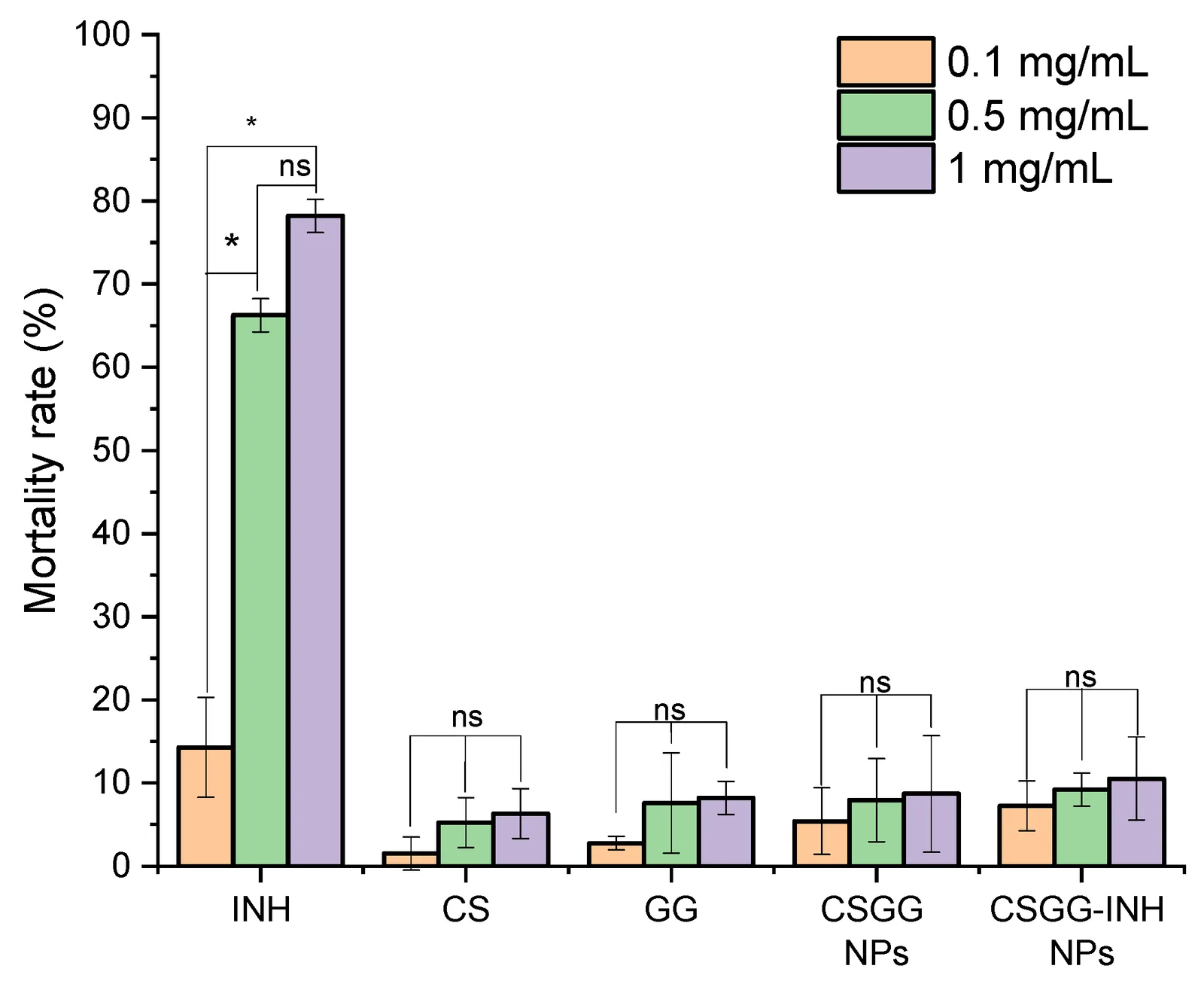
Mortality rate of Artemia salina nauplii after 24-h exposure to isoniazid, chitosan, gellan gum, CSGG nanoparticles, and CSGG–INH at the concentrations indicated. “*” indicates *p* < 0.05; ns = not significant.

In order to study the toxicity of the produced nanoparticles, survival tests were conducted on Artemia salina nauplii [62,63]. In this study, Artemia salina nauplii were treated with various concentrations (0.1–1 mg/mL) of isoniazid, chitosan, gellan gum, CSGG NPs, and CSGG−INH NPs. Cell viability was determined 24 h after treatment. The results are presented in Figure 11.

As shown in Figure 11, the most pronounced toxic effect was observed for isoniazid. As the INH concentration increased from 0.1 to 1 mg/mL, the mortality rate of Artemia salina increased from 14 to 78%. For CS, the mortality rate was 1.5–6.2%, and for GG, it ranged from 2.8 to 8.3%. A similar trend was observed for empty CS-GG nanoparticles: the mortality rate ranged from 5.4 to 8.6%. Despite the presence of isoniazid, the mortality rates of Artemia salina nauplii exposed to CSGG–INH NPs were 7.2%, 8.9%, and 10.5% at concentrations of 0.1, 0.5, and 1 mg/mL, respectively. Furthermore, at all concentrations tested, the mortality rates for INH-containing nanoparticles remained significantly lower than those for free INH [64,65]. Thus, isoniazid exhibited moderate cytotoxicity (LC_50_ = 352 µg/mL), whereas the other components of the formulation and the nanoparticles themselves showed no toxicity toward Artemia salina (LC_50_ > 1000 µg/mL) [66,67]. Notably, the reduced mortality observed with CSGG–INH NPs compared with free INH suggests that incorporation of INH into the polymer matrix may reduce its acute toxic effects.

## 4. Conclusions

In this study, mucoadhesive nanoparticles of a chitosan-gellan gum polyelectrolyte complex loaded with isoniazid (CSGG–INH NP) were successfully synthesized and characterized to evaluate their potential as carriers for pulmonary delivery of isoniazid. The optimal formulation was determined by varying the amount of chitosan and gellan gum and the pH, and nanoparticles with promising physicochemical properties, such as a mean particle size of 178 ± 7 nm, a polydispersity index of 0.269 ± 0.025, a positive ζ-potential of 22 ± 3 mV, a loading of isoniazid up to 61%, and a yield of nanoparticles of 26%, were obtained. TEM and NTA analysis confirmed the successful formation of spherical nanoparticles, and FTIR, DSC, and TGA analysis confirmed the successful formation of chitosan-gellan nanoparticles and the effective incorporation of isoniazid. The resulting nanoparticles showed a high degree of mucoadhesion and stayed adhered to ovine lung tissue even under dynamic washing. The release profile in vitro in phosphate buffer solution (PBS, pH 7.4) was biphasic, with a fast initial release and sustained release, highlighting the polymer matrix’s capacity to provide prolonged drug release. In addition, CSGG–INH nanoparticles completely eliminated the growth of H37Rv *Mycobacterium tuberculosis* in the MGIT system, indicating that the biologically active isoniazid was released during the entire incubation period. The toxicity assessment using Artemia salina nauplii showed that free INH exhibited moderate toxicity (LC_50_ = 352 μg/mL), whereas CSGG–INH nanoparticles caused low mortality across the concentration range studied, indicating a reduction in toxic effects following the incorporation of INH into the nanoparticle matrix. In conclusion, the chitosan-gellan gum polyelectrolyte complex nanoparticles, with their high mucoadhesion, sustained drug release, and preserved antimycobacterial activity, suppor further investigation as potential nanocarriers for the pulmonary delivery of isoniazid. These findings provide a basis for future studies evaluating aerosol performance, pulmonary deposition, pharmacokinetics, safety, and therapeutic efficacy in appropriate in vitro and in vivo models.

## 5. Patents

The results presented in this study are protected by a Republic of Kazakhstan Utility Model Patent No. 12041, entitled “Method for obtaining chitosan and gellan nanoparticles for loading the anti-tuberculosis drug isoniazid”.

## Figures and Tables

**Table 1:** Effect of chitosan-to-gellan gum ratio and pH on the formation of chitosan–gellan gum nanoparticles.

pH	Mass Ratio of CS:GG	NPs	Average Diameter (nm)	Average PDI	Average Zeta Potential (mV)	Yield of NPs (%)
2	9:1	C9G1	343 ± 12	0.354 ± 0.012	24 ± 1	27
	8:2	C8G2	254 ± 5	0.258 ± 0.014	25 ± 2	25
	7:3	C7G3	274 ± 1	0.235 ± 0.014	22 ± 2	37
	6:4	C6G4	266 ± 3	0.211 ± 0.060	17 ± 2	58
	5:5	C5G5	308 ± 5	0.252 ± 0.014	19 ± 1	52
	4:6	C4G6	214 ± 15	0.270 ± 0.012	28 ± 2	63
	3:7	C3G7	358 ± 6	0.234 ± 0.039	25 ± 1	55
	2:8	C2G8	341 ± 2	0.431 ± 0.092	19 ± 3	60
	1:9	C1G9	290 ± 1	0.042 ± 0.020	−10 ± 1	60
5	9:1	C9G1-pH5	522 ± 16	0.597 ± 0.081	23 ± 2	28
	8:2	C8G2-pH5	619 ± 16	0.638 ± 0.084	29 ± 2	18
	7:3	C7G3-pH5	324 ± 26	0.466 ± 0.063	25 ± 3	35
	6:4	C6G4-pH5	342 ± 19	0.488 ± 0.201	25 ± 2	42
	5:5	C5G5-pH5	302 ± 6	0.225 ± 0.031	17 ± 2	65
	4:6	C4G6-pH5	249 ± 8	0.271 ± 0.006	16 ± 0.3	68
	3:7	C3G7-pH5	199 ± 5	0.061 ± 0.027	13 ± 2	70
	2:8	C2G8-pH5	221 ± 10	0.134 ± 0.038	−26 ± 2	73
	1:9	C1G9-pH5	124 ± 1	0.252 ± 0.020	−18 ± 1	30

All values are presented as mean ± standard deviation (n = 3).

**Table 2:** Effect of isoniazid loading on the formation of chitosan–gellan gum nanoparticles.

NPs	Average Diameter (nm)	Average PDI	Average Zeta Potential (mV)	EE (%)	LD (%)	Yield of NPs (%)
C4G6-INH	178 ± 7	0.269 ± 0.025	22 ± 3	32	61	26
C2G8-INH-pH5	185 ± 3	0.118 ± 0.078	−15 ± 1	18	22	42

All values are presented as mean ± S.D. (n = 3).

## Data Availability

The original contributions presented in this study are included in the article. Further inquiries can be directed to the corresponding author.

## Supplementary Materials

The following supporting information can be downloaded at: https://www.mdpi.com/article/10.3390/polym18172139/s1, Video S1: “Representative Nanoparticle Tracking Analysis Video Demonstrating the Brownian Motion of CSGG Nanoparticles”; Video S2: “Representative Nanoparticle Tracking Analysis Video Demonstrating the Brownian Motion of CSGG–INH Nanoparticles”.

## Abbreviations

The following abbreviations are used in this manuscript:

TB	Tuberculosis
MTB	*Mycobacterium tuberculosis*
CS	Chitosan
GG	Gellan Gum
NPs	Nanoparticles
INH	Isoniazid
PDI	Polydispersity Index
EE	Encapsulation Efficiency
TGA	Thermogravimetric Analysis
DSC	Differential Scanning Calorimetry
FTIR	Fourier-Transform Infrared Spectroscopy
ANOVA	Analysis of Variance
FITC	Fluorescein Isothiocyanate
TEM	Transmission electron microscopy
MGIT	Mycobacteria Growth Indicator Tube
PBS	Phosphate buffer solution
NTA	Nanoparticle Tracking Analysis
CFU	Colony-forming units

## References

[1] WHO (2025). Global Tuberculosis Report 2025.

[2] World Health Organization (2008). Implementing the WHO Stop TB Strategy: A Handbook for National Tuberculosis Control Programmes.

[3] Dobbs T.E., Webb R.M. (2017). Chemotherapy of Tuberculosis. Microbiol. Spectr..

[4] Badrinath M., Chen R.J., John S. (2024). Isoniazid Toxicity.

[5] dos Santos A.M., Carvalho S.G., Meneguin A.B., Sábio R.M., Gremião M.P.D., Chorilli M. (2021). Oral delivery of micro/nanoparticulate systems based on natural polysaccharides for intestinal diseases therapy: Challenges, advances and future perspectives. J. Control. Release.

[6] Sirisha V.L., D’Souza J.S. (2016). Polysaccharide-Based Nanoparticles as Drug Delivery Systems. Marine OMICS.

[7] Picone C.S.F., Cunha R.L. (2013). Chitosan–gellan electrostatic complexes: Influence of preparation conditions and surfactant presence. Carbohydr. Polym..

[8] Verma A., Kumar P., Rastogi V., Mittal P. (2021). Preparation and evaluation of polymeric beads composed of Chitosan–Gellan Gum–Gum Ghatti/-Gum Karaya polyelectrolyte complexes as polymeric carrier for enteric sustained delivery of Diclofenac sodium. Future J. Pharm. Sci..

[9] Potaś J., Szymańska E., Basa A., Hafner A., Winnicka K. (2020). Tragacanth Gum/Chitosan Polyelectrolyte Complexes-Based Hydrogels Enriched with Xanthan Gum as Promising Materials for Buccal Application. Materials.

[10] Mahajan H.S., Patil P.P. (2017). In situ cross Linked Chitosan-Gellan Gum Polyelectrolyte Complex Based Nanogels Containing Curcumin for Delivery to Cancer Cells. Indian J. Pharm. Educ. Res..

[11] Vehlow D., Schmidt R., Gebert A., Siebert M., Lips K., Müller M. (2016). Polyelectrolyte Complex Based Interfacial Drug Delivery System with Controlled Loading and Improved Release Performance for Bone Therapeutics. Nanomaterials.

[12] Le H.V., Le Cerf D. (2022). Colloidal Polyelectrolyte Complexes from Hyaluronic Acid: Preparation and Biomedical Applications. Small.

[13] de Oliveira A.C., Vilsinski B.H., Bonafé E.G., Monteiro J.P., Kipper M.J., Martins A.F. (2019). Chitosan content modulates durability and structural homogeneity of chitosan-gellan gum assemblies. Int. J. Biol. Macromol..

[14] Lankalapalli S., Kolapalli V.R.M. (2009). Polyelectrolyte complexes: A review of their applicability in drug delivery technology. Indian J. Pharm. Sci..

[15] Kumar S., Kaur P., Bernela M., Rani R., Thakur R. (2016). Ketoconazole encapsulated in chitosan-gellan gum nanocomplexes exhibits prolonged antifungal activity. Int. J. Biol. Macromol..

[16] Cardoso V.M.D.O., Brito N.A.P.D., Ferreira N.N., Boni F.I., Ferreira L.M.B., Carvalho S.G., Gremião M.P.D. (2021). Design of mucoadhesive gellan gum and chitosan nanoparticles intended for colon-specific delivery of peptide drugs. Colloids Surf. A Physicochem. Eng. Asp..

[17] Carvalho S.G., Haddad F.F., dos Santos A.M., Scarim C.B., Ferreira L.M.B., Meneguin A.B., Chorilli M., Gremião M.P.D. (2024). Chitosan surface modification modulates the mucoadhesive, permeation and anti-angiogenic properties of gellan gum/bevacizumab nanoparticles. Int. J. Biol. Macromol..

[18] Ferreira L.M.B., dos Santos A.M., Boni F.I., dos Santos K.C., Robusti L.M.G., de Souza M.P.C., Ferreira N.N., Carvalho S.G., Cardoso V.M.O., Chorilli M. (2020). Design of chitosan-based particle systems: A review of the physicochemical foundations for tailored properties. Carbohydr. Polym..

[19] Carvalho S.G., dos Santos A.M., Silvestre A.L.P., Meneguin A.B., Ferreira L.M.B., Chorilli M., Gremião M.P.D. (2021). New insights into physicochemical aspects involved in the formation of polyelectrolyte complexes based on chitosan and dextran sulfate. Carbohydr. Polym..

[20] Gomes D., Batista-Silva J.P., Sousa A., Passarinha L.A. (2023). Progress and opportunities in Gellan gum-based materials: A review of preparation, characterization and emerging applications. Carbohydr. Polym..

[21] Zacaron T.M., Silva M.L.S.E., Costa M.P., Silva D.M.E., Silva A.C., Apolônio A.C.M., Fabri R.L., Pittella F., Rocha H.V.A., Tavares G.D. (2023). Advancements in Chitosan-Based Nanoparticles for Pulmonary Drug Delivery. Polymers.

[22] Shah S., Maheshwari H., Soniwala M., Chavda J. (2020). Pulmonary Delivery of Linezolid Nanoparticles for Treatment of Tuberculosis: Design, Development, and Optimization. J. Pharm. Innov..

[23] Motiei M., Pleno de Gouveia L., Šopík T., Vícha R., Škoda D., Císař J., Khalili R., Domincová Bergerová E., Münster L., Fei H. (2021). Nanoparticle-Based Rifampicin Delivery System Development. Molecules.

[24] Cook M.T., Tzortzis G., Charalampopoulos D., Khutoryanskiy V.V. (2011). Production and Evaluation of Dry Alginate-Chitosan Microcapsules as an Enteric Delivery Vehicle for Probiotic Bacteria. Biomacromolecules.

[25] Ge Y., Zhang Y., He S., Nie F., Teng G., Gu N. (2009). Fluorescence Modified Chitosan-Coated Magnetic Nanoparticles for High-Efficient Cellular Imaging. Nanoscale Res. Lett..

[26] Cetin F.N., Cernochova Z., Vermeersch E., Herynek V., Groborz O., Pavlova E., Slouf M., Hruby M., Mignon A., Hoogenboom R. (2025). Dual thermo- and ROS-responsive triblock copolymers as 19F MRI tracers for functional nanoparticles and hydrogels. Eur. Polym. J..

[27] Slouf M., Sikorova P., Pavlova E., Swietek M., Lartigue L., Skoupy R., Krzyzanek V. (2025). 4D-STEM-in-SEM: Changing an SEM Microscope to a User-friendly Powder Electron Diffractometer. Microsc. Microanal..

[28] Kostka L., Kotrchová L., Šubr V., Libánská A., Ferreira C.A., Malátová I., Lee H.J., Barnhart T.E., Engle J.W., Cai W. (2020). HPMA-based star polymer biomaterials with tuneable structure and biodegradability tailored for advanced drug delivery to solid tumours. Biomaterials.

[29] Phuong Ta L., Bujna E., Kun S., Charalampopoulos D., Khutoryanskiy V.V. (2021). Electrosprayed mucoadhesive alginate-chitosan microcapsules for gastrointestinal delivery of probiotics. Int. J. Pharm..

[30] Dzodanu E.G., Afrifa J., Acheampong D.O., Dadzie I. (2019). Diagnostic Yield of Fluorescence and Ziehl-Neelsen Staining Techniques in the Diagnosis of Pulmonary Tuberculosis: A Comparative Study in a District Health Facility. Tuberc. Res. Treat..

[31] Lai P., Daear W., Löbenberg R., Prenner E.J. (2014). Overview of the preparation of organic polymeric nanoparticles for drug delivery based on gelatine, chitosan, poly(d,l-lactide-co-glycolic acid) and polyalkylcyanoacrylate. Colloids Surf. B Biointerfaces.

[32] Hu Y., Yang T., Hu X. (2011). Novel polysaccharides-based nanoparticle carriers prepared by polyelectrolyte complexation for protein drug delivery. Polym. Bull..

[33] Montero N., Alhajj M.J., Sierra M., Oñate-Garzon J., Yarce C.J., Salamanca C.H. (2020). Development of Polyelectrolyte Complex Nanoparticles-PECNs Loaded with Ampicillin by Means of Polyelectrolyte Complexation and Ultra-High Pressure Homogenization (UHPH). Polymers.

[34] Margossian K.O., Brown M.U., Emrick T., Muthukumar M. (2022). Coacervation in polyzwitterion-polyelectrolyte systems and their potential applications for gastrointestinal drug delivery platforms. Nat. Commun..

[35] Ahmed R., Hira N.U.A., Fu Z., Wang M., Halepoto A., Khanal S., Iqbal S., Mahar H., Cohen Stuart M.A., Guo X. (2021). Control and Preparation of Quaternized Chitosan and Carboxymethyl Chitosan Nanoscale Polyelectrolyte Complexes Based on Reactive Flash Nanoprecipitation. ACS Omega.

[36] Johnson B.K., Prud’homme R.K. (2003). Flash NanoPrecipitation of Organic Actives and Block Copolymers using a Confined Impinging Jets Mixer. Aust. J. Chem..

[37] Tazhbayev Y.M., Cheung S.Y., Galiyeva A.R., Zhumagaliyeva T.S., Daribay A.T. (2026). Method for Obtaining Chitosan and Gellan Nanoparticles for Loading the Anti-Tuberculosis Drug Isoniazid. Patent for Utility Model of Republic of Kazakhstan.

[38] Sogias I.A., Williams A.C., Khutoryanskiy V.V. (2008). Why is Chitosan Mucoadhesive?. Biomacromolecules.

[39] Galiyeva A., Tazhbayev Y., Zhumagaliyeva T., Karimova B., Tabriz N., Khutoryanskiy V.V. (2025). Synthesis and Investigation of Albumin Nanoparticles Loaded With Anti-Tuberculosis Drug Isoniazid. Biopolymers.

[40] Ahalwat S., Bhatt D.C., Rohilla S., Jogpal V., Sharma K., Virmani T., Kumar G., Alhalmi A., Alqahtani A.S., Noman O.M. (2023). Mannose-Functionalized Isoniazid-Loaded Nanostructured Lipid Carriers for Pulmonary Delivery: In Vitro Prospects and In Vivo Therapeutic Efficacy Assessment. Pharmaceuticals.

[41] Zhang M., Chen W., Ju Y., Zhao H., Wang C. (2023). Polymer–Protein Nanovaccine Synthesized via Reactive Self-Assembly with Potential Application in Cancer Immunotherapy: Physicochemical and Biological Characterization In Vitro and In Vivo. Macromol. Rapid Commun..

[42] Liu L., Yu W., Seitsonen J., Xu W., Lehto V.-P. (2022). Correct Identification of the Core-Shell Structure of Cell Membrane-Coated Polymeric Nanoparticles. Chem.—Eur. J..

[43] Konne J.L., Benneth S.O. (2014). Synthesis and Characterization of Chitosan-Copper complex using Shells of Fresh Water Shrimps and Salt Water Crabs in Rivers State as Raw Materials. J. Niger. Environ. Soc..

[44] Silva-Correia J., Oliveira J.M., Caridade S.G., Oliveira J.T., Sousa R.A., Mano J.F., Reis R.L. (2011). Gellan gum-based hydrogels for intervertebral disc tissue-engineering applications. J. Tissue Eng. Regen. Med..

[45] Modrogan C., Pandele A.M., Bobirică C., Dobrotǎ D., Dăncilă A.M., Gârleanu G., Orbuleţ O.D., Borda C., Gârleanu D., Orbeci C. (2020). Synthesis, Characterization and Sorption Capacity Examination for a Novel Hydrogel Composite Based on Gellan Gum and Graphene Oxide (GG/GO). Polymers.

[46] Markarian S.A., Papanyan Z.K., Shahinyan G.A. (2020). Volumetric, UV–Vis and FT IR Studies of Isoniazid in Diethylsulfoxide Solutions. J. Solut. Chem..

[47] Ngilirabanga J.B., Aucamp M., Pires Rosa P., Samsodien H. (2020). Mechanochemical Synthesis and Physicochemical Characterization of Isoniazid and Pyrazinamide Co-crystals With Glutaric Acid. Front. Chem..

[48] Cheung S.-Y., Galiyeva A., Tazhbayev Y., Zhumagaliyeva T., Bardadym Y., Aseyev V. (2026). Mucoadhesive Chitosan–Gellan Gum Nanoparticles for Rifampicin Delivery: Taguchi Optimization and In Vitro Release Behavior. Pharmaceutics.

[49] Gupta K.C., Jabrail F.H. (2007). Effect of molecular weight and degree of deacetylation on controlled release of isoniazid from chitosan microspheres. Polym. Adv. Technol..

[50] Ferrero F., Periolatto M. (2012). Antimicrobial Finish of Textiles by Chitosan UV-Curing. J. Nanosci. Nanotechnol..

[51] Galiyeva A., Daribay A., Tabriz N., Tazhbayev Y. (2025). Production of Polylactide Nanoparticles Loaded With Isoniazid and Vitamin C: A Promising Candidate for the Treatment of Resistant Forms of Tuberculosis. J. Polym. Sci..

[52] Pedreiro L.N., Ferreira Cury B.S., Daflon Gremião M.P. (2016). Mucoadhesive Nanostructured Polyelectrolyte Complexes as Potential Carrier to Improve Zidovudine Permeability. J. Nanosci. Nanotechnol..

[53] Galiyeva A., Daribay A., Zhumagaliyeva T., Zhaparova L., Sadyrbekov D., Tazhbayev Y. (2023). Human Serum Albumin Nanoparticles: Synthesis, Optimization and Immobilization with Antituberculosis Drugs. Polymers.

[54] Ahmad K., Zhang Y., Chen P., Yang X., Hou H. (2024). Chitosan interaction with stomach mucin layer to enhances gastric retention and mucoadhesive properties. Carbohydr. Polym..

[55] Ahmad N. (2017). Rasagiline-encapsulated chitosan-coated PLGA nanoparticles targeted to the brain in the treatment of parkinson’s disease. J. Liq. Chromatogr. Relat. Technol..

[56] Yessentayeva N.A., Galiyeva A.R., Daribay A.T., Sadyrbekov D.T., Moustafine R.I., Tazhbayev Y.M. (2024). Optimization of Polylactide-Co-Glycolide-Rifampicin Nanoparticle Synthesis, In Vitro Study of Mucoadhesion and Drug Release. Polymers.

[57] Thai H., Thuy Nguyen C., Thi Thach L., Thi Tran M., Duc Mai H., Thi Thu Nguyen T., Duc Le G., Van Can M., Dai Tran L., Long Bach G. (2020). Characterization of chitosan/alginate/lovastatin nanoparticles and investigation of their toxic effects in vitro and in vivo. Sci. Rep..

[58] Tazhbayev Y.M., Galiyeva A.R., Syrymova U.Y., Zhaparova L.Z., Zhumagaliyeva T.S. (2026). Rifampicin Loaded Chitosan-Based Nanoparticles: Optimization, Characterization, and Mucoadhesion. EURASIAN J. Chem..

[59] Ghosh R., Mondal S., Mukherjee D., Adhikari A., Ahmed S.A., Alsantali R.I., Khder A.S., Altass H.M., Moussa Z., Das R. (2022). Oral drug delivery using a polymeric nanocarrier: Chitosan nanoparticles in the delivery of rifampicin. Mater. Adv..

[60] Bayer I.S. (2023). Controlled Drug Release from Nanoengineered Polysaccharides. Pharmaceutics.

[61] Wu I.Y., Bala S., Škalko-Basnet N., di Cagno M.P. (2019). Interpreting non-linear drug diffusion data: Utilizing Korsmeyer-Peppas model to study drug release from liposomes. Eur. J. Pharm. Sci..

[62] Bhoopathy S., Inbakandan D., Thirugnanasambandam R., Kumar C., Sampath P., Bethunaickan R., Raguraman V., Vijayakumar G.K. (2021). A comparative study on chitosan nanoparticle synthesis methodologies for application in aquaculture through toxicity studies. IET Nanobiotechnol..

[63] Rajabi S., Ramazani A., Hamidi M., Naji T. (2015). Artemia salina as a model organism in toxicity assessment of nanoparticles. DARU J. Pharm. Sci..

[64] Mukhtar M., Csaba N., Robla S., Varela-Calviño R., Nagy A., Burian K., Kókai D., Ambrus R. (2022). Dry Powder Comprised of Isoniazid-Loaded Nanoparticles of Hyaluronic Acid in Conjugation with Mannose-Anchored Chitosan for Macrophage-Targeted Pulmonary Administration in Tuberculosis. Pharmaceutics.

[65] Bhandari R., Singh M., Jindal S., Kaur I.P. (2021). Toxicity studies of highly bioavailable isoniazid loaded solid lipid nanoparticles as per Organisation for Economic Co-operation and Development (OECD) guidelines. Eur. J. Pharm. Biopharm..

[66] Meyer B., Ferrigni N., Putnam J., Jacobsen L., Nichols D., McLaughlin J. (1982). Brine Shrimp: A Convenient General Bioassay for Active Plant Constituents. Planta Med..

[67] Clarkson C., Maharaj V.J., Crouch N.R., Grace O.M., Pillay P., Matsabisa M.G., Bhagwandin N., Smith P.J., Folb P.I. (2004). In Vitro Antiplasmodial Activity of Medicinal Plants Native to or Naturalised in South Africa. J. Ethnopharmacol..

